# Geomicrobiological characterization of the evaporitic ecosystem in the hypersaline lake Laguna Verde (Andean Puna, Northwestern Argentina)

**DOI:** 10.1002/ece3.10931

**Published:** 2024-02-12

**Authors:** L. A. Saona, P. G. Villafañe, D. Carrizo, C. Cónsole Gonella, R. F. Néspolo, M. E. Farías

**Affiliations:** ^1^ Facultad de Química y Biología Universidad de Santiago de Chile (USACH) Santiago Chile; ^2^ Millennium Nucleus of Patagonian Limit of Life (LiLi) Valdivia Chile; ^3^ Millennium Institute for Integrative Biology (iBio) Santiago Chile; ^4^ Instituto Superior de Correlación Geológica (INSUGEO), CONICET‐UNT Tucumán Argentina; ^5^ GIUV2016‐303, Department of Botany and Geology Universitat de València València España; ^6^ Centro de Astrobiología Instituto Nacional de Técnica Aeroespacial Madrid España; ^7^ Instituto de Ciencias Ambientales y Evolutivas Universidad Austral de Chile Valdivia Chile; ^8^ PUNABIO S.A. Campus USP‐T San Pablo Argentina

**Keywords:** cyanobacteria, dome‐shaped structures, evaporites, extremophiles, geomicrobiology, gypsum

## Abstract

Laguna Verde's dome‐shaped structures are distinctive formations within the Central Andes, displaying unique geomicrobiological features. This study represents a pioneering investigation into these structures, assessing their formation, associated taxa, and ecological significance. Through a multifaceted approach that includes chemical analysis of the water body, multiscale characterization of the domes, and analysis of the associated microorganisms, we reveal the complex interplay between geology and biology in this extreme environment. The lake's alkaline waters that are rich in dissolved cations and anions such as chloride, sodium sulfate, and potassium, coupled with its location at the margin of the Antofalla salt flat, fed by alluvial fans and hydrothermal input, provide favorable conditions for mineral precipitation and support for the microorganism's activity. Laguna Verde's dome‐shaped structures are mainly composed of gypsum and halite, displaying an internal heterogeneous mesostructure consisting of three zones: *microcrystalline*, *organic* (orange and green layers), and *crystalline*. The green layer of the *organic zone* is predominantly composed of Proteobacteria, Bacteroidetes, and Cyanobacteria, while the orange layer is mostly inhabited by Cyanobacteria. The results of the study suggest that oxygenic photosynthesis performed by Cyanobacteria is the main carbon fixation pathway in the microbial community, supported by carbon isotopic ratios of specific biomarkers. This finding highlights the important role played by Cyanobacteria in this ecosystem.

## Graphical abstract

This study provides the first comprehensive geomicrobiological analysis of the dome‐shaped structures in Laguna Verde, a crucial Andean Microbial Ecosystem (AME) in the Central Andes. The research unveils the structure's diverse micro‐environments and the associated microorganisms, primarily Cyanobacteria, which play a significant role in the ecosystem's carbon fixation through oxygenic photosynthesis. This investigation into Laguna Verde's unique gypsum evaporitic microbial ecosystems (GEMEs) contributes critical insights into similar records throughout Earth's history, thus, aiding studies of life's origin and astrobiology.

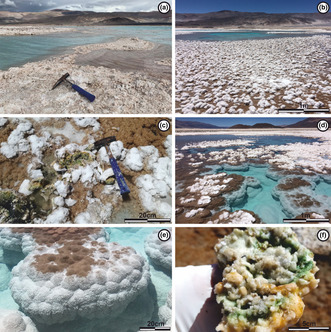

## 1 INTRODUCTION

Microorganisms in marine, lagoon, lake, or riverine environments directly influence the geochemical processes that form the sedimentary record of our planet (Dupraz et al., 2009; Farías et al., 2014; Gerdes, 2007). They can also regulate the stabilization of sediments (Paterson, 1994), influence the precipitation or dissolution of minerals (Visscher & Stolz, 2005), and control their composition and morphology (Braissant et al., 2004), as well as the macrostructure and microstructure (Burne & Moore, 1987; Dupraz et al., 2009; Reid et al., 2000) in the development of organo‐sedimentary structures (microbialites).

The most studied lithifying microbial ecosystems have a carbonatic composition with calcium, calcium–magnesium (such as Mg‐calcite and dolomite), and magnesium (magnesite). However, in some systems, the microbial communities are mostly associated with gypsum and halite, classified as gypsum evaporitic microbial ecosystems (GEMEs) (Vignale et al., 2021). At present, limited information is known about the role of microorganisms in gypsum precipitation (Farías et al., 2014; Fernandez et al., 2016; Herrero, 2019). Nevertheless, in recent years, microorganism ecosystems associated with gypsum deposits have gained great importance in relation to studies linked with the origin of life and astrobiology, due to their high potential to preserve biosignatures (Allwood et al., 2013; Phillips et al., 2023; Stivaletta et al., 2010).

GEMEs have been reported in shores of high‐salinity lakes in the Central Andes region, listed as (i) biofilms attached to the surface of minerals, where microorganisms extract water from them (Vignale et al., 2021); (ii) endolithic communities (endoevaporites), where minerals provide protection against desiccation and UV‐B radiation but allow photosynthetically active radiation (Vignale et al., 2021); or (iii) organo‐sedimentary structures (microbialites), formed by the dynamic interaction between microorganisms and the environment (Herrero, 2019; Phillips et al., 2023). Given its expanse and distinctive dome‐shaped structures that inhabit the lake, Laguna Verde stands out as a notable ‘endoevaporitic’ deposit in the Central Andes (see Vignale et al., 2021). However, these structures have not been studied yet in depth.

This study aims to conduct the first analysis of the microbial community, key metabolic pathways, and lipid biomarkers in the dome‐shaped structures of Laguna Verde (Central Andes). For this purpose, we performed a complete microbial (taxonomic and metagenomic) and biomarker analysis of the mats, together with petrographic determination of the domes and the chemistry of the host water body. In this way, we provide a characterization of these gypsum evaporitic microbial ecosystems, that can be used to evaluate and interpret modern or fossil analogous deposits in the geological record and shed light on aspects such as gypsum precipitation, the sulfur cycle, or the microbial biodiversity associated with these environments.

## 2 GEOGRAPHICAL LOCATION AND GEOLOGICAL CONTEXT

**FIGURE 1:**
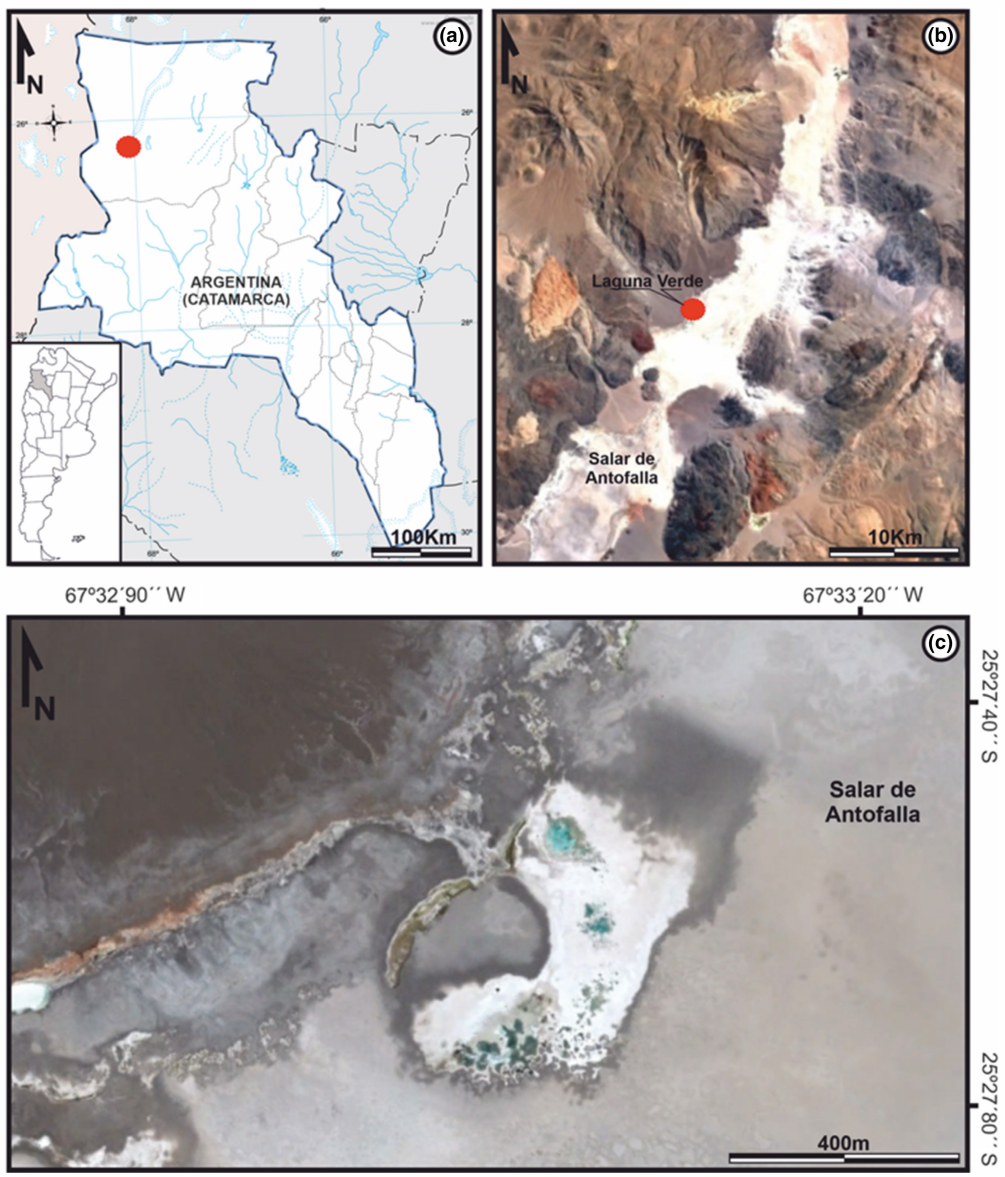
Laguna Verde's location and geographical overview. (a) Location of the working area (red circle) in the province of Catamarca, Argentina. (b) Satellite image of the Salar de Antofalla basin showing the position of Laguna Verde (red circle). (c) Satellite image showing the body of water called Laguna Verde, located on the western edge of the salt flat.

Laguna Verde is a lake located within the Salar de Antofalla salt flat (Figure 1a,b) (25°28′45.73″ S, 67°33′17.72″ W), at an elevation of 3343 m above sea level (masl) (Vignale et al., 2021). It belongs to the Argentine Puna region, an elevated area in the Central Andes (3700 masl) with a steep local relief caused by contractional tectonics, volcanic activity, and slow erosion due to an arid climate (Alonso & Rojas, 2020; Kraemer et al., 1999).

The Puna region is characterized by sedimentary basins with thick sequences of continental evaporites (water‐soluble sedimentary mineral deposit that results from concentration and crystallization by evaporation from an aqueous solution), developed during the Miocene epoch to recent periods, intercalated with late Eocene to Holocene clastic deposits (Alonso et al., 2006; Kraemer et al., 1999). The endorheic drainage of these basins, combined with an arid to semiarid climate and interior drainage, has resulted in the formation of lacustrine environments such as salt lakes and wetlands (Alonso & Rojas, 2020).

The conditions in these lacustrine environments, such as high levels of solar irradiance (with incident UV‐B flux reaching 10 W m^−2^) and surface solar radiation (levels of approximately 310 W m^−2^), daily insolation of 6.6 k W h m^−2^ day^−1^, average annual temperatures of 4.7–5.3°C, large daily and seasonal temperature ranges (up to 30°C) resulting in daily water freezing and thawing, low levels of nutrient availability, and high concentrations of heavy metals and metalloids (such as As) and other toxic elements harmful to human life, make these lacustrine environments some of the most extreme on the planet (Albarracín et al., 2016; Farías et al., 2011, 2013, 2014; Saona et al., 2020).

The Salar de Antofalla basin is located in the Puna northwest region, between the Andes range and Sierra de Calalaste (Catamarca, Argentina). This basin is characterized by a basement composed of Precambrian metamorphic rocks and Paleozoic plutonic and sedimentary rocks, which are overlain by Cretaceous and Paleogene sediments. Followed in the stratigraphic sequence by thick sequences of continental evaporites formed during the Miocene epoch to recent periods, unconformably overlie by late Eocene–Miocene clastic deposits (Alonso et al., 2006; Kraemer et al., 1999).

Salar de Antofalla is approximately 140 km long and between 4 and 10 km wide (Voss, 2002). Characterized by internal drainage and temporary surface flow conditions, the evaporitic deposits that make up the salt flat consist essentially of chlorides and sulfates of sodium, calcium, potassium, magnesium, and lithium (Hongn & Seggiaro, 2001).

The brines contained in Salar de Antofalla originate from the contribution of thermal waters and the infiltration of meteoric waters, which leach the surrounding rocks and concentrate by evaporation. In the salt flat aquifers, the brines typically show an increase in concentration from the margins to the core (Hongn & Seggiaro, 2001).

Interactions between hypersaline waters and meteoric water control the precipitation and dissolution of minerals such as carbonates. In addition, the discharge of groundwater into the salt flat supplies nutrients and dissolved ions, creating a lower‐salinity environment that has an important effect on the living conditions of microorganisms (Gomez et al., 2014; Houston et al., 2011; Suosaari et al., 2018; Warden et al., 2019). These interactions and the unique conditions present in the salt flat can have an impact on the microbial communities and metabolic pathways present in the environment.

## 3 METHODOLOGY

### 3.1 Sample collection

In the first stage, a geological survey of the deposit and its surroundings was carried out. For this, satellite images obtained from SAS Planet software were used as a basis. On these images, the lithological information provided by the geological chart HG 2469‐IV Antofalla was digitized using ArcGIS software. Each lithological unit or rock type is classified according to the legend available in the geological chart. As a result, a geological map of the work area was obtained at a scale of 1:80,000 (Figure S1).

In 2019, three sampling campaigns (January, August, and October) were conducted for water ion chromatography and metagenomic analyses. For the metagenomic analyses, samples were collected from the green and orange layers within the dome‐shaped structures. Using a sterile scalpel, each layer was carefully separated to obtain three independent samples from endoevaporitic gypsum crust, resulting in three samples from the green layer and three from the orange layer. These samples were stored in RNAlater® solution (Thermo Fisher Scientific, USA) at 4°C in the dark and processed within 1 week in the laboratory.

After this, during the year 2020, a seasonal survey of Laguna Verde was carried out. It was initiated with the delimitation of the study area, the geomorphological description of the lake, and the location of the structures of interest. Subsequently, in situ measurements of pH, electrical conductivity, dissolved oxygen, temperature, and depth of the water body were carried out. For the lipid biomarkers analysis, a biogeochemical sampling campaign was undertaken in January 2020, where two samples from an endoevaporitic gypsum crust (green layer, G.E; orange layer, O.E) were obtained. Samples of about 3 g were collected with solvent‐cleaned (dichloromethane and methanol) stainless‐steel tweezers, spoon, and scraper and transferred to polyethylene containers, where they were kept cold (~4°C) until biogeochemical analysis in the laboratory.

The chemical composition of the water was determined by ion chromatography, carried out by the company Grupo Induser SRL (Salta, Argentina). Water samples were taken during two seasons of the year – summer (January) and winter (August) – to monitor potential seasonal variations. These samples were stored at the Instituto Superior de Correlación Geológica (INSUGEO, CONICET, Argentina), in a cool, dark environment until analysis to prevent any changes in the sample composition. Prior to conducting ion chromatography, the water was filtered using 0.2–0.45‐micrometer pore‐sized filters to eliminate suspended particles before measurements. Additionally, if necessary, degassing was performed using a vacuum apparatus to remove bubbles that might interfere with the analysis.

The methodologies used for quantification are as follows: determination of anions (chloride, sulfate, and nitrate) and cations (potassium, magnesium, calcium, and sodium) by ion chromatography, as per ISO 14911:1998. Nitrite (SM 4500‐NO2), nitrate (SM 4500‐NO3), phosphorus (SM 4500‐P), and ammonia (SM 4500‐NH3) concentrations were determined following the Standard Methods for the Examination of Water and Wastewater (Rice et al., 2017). Chemical Oxygen Demand (COD, SM 5220) and Biological Oxygen Demand (BOD, SM 5210) were determined using titrimetric methods. These involve the oxidation of organic matter in the sample. Manganese and iron concentrations were determined by EPA 3015, an open vessel microwave‐assisted acid digestion method. Total hardness (SM 2340) was determined by a complexometric titration method.

### 3.2 Characterization of dome‐shaped structures: multiscale analysis and mineral fraction assessment

Dome‐shaped structures were described following a traditional multiscale approach (Shapiro, 2000; Vennin et al., 2015). This was focused on the separate characterization of the megastructure (i.e., large‐scale features of microbialite bed), macrostructure (i.e., gross form of microbialite bodies with typical dimensions from decimeter to meter scale), mesostructure (i.e., internal textures of macrostructural elements visible to naked eye), and microstructure (i.e., microscopic fabrics observed under petrographic analysis).

External morphology (megastructure and macrostructure) was established using field data, whereas internal morphology (mesostructure and microstructure) was examined using a Leica DMS 100 binocular loupe and a petrographic microscope equipped with a Leica DFC 420 camera. Mineral fractions of the dome‐shape structures were analyzed using a Malvern PANalytical Empyrean x‐ray diffractometer, with X'Celerator detector and Cu tube. The scan rate was 0.5°/min, under a voltage of 40 kv and current of 30 mA. The dried and ground samples were scanned in the 2·ϴ‐diffraction angle from 5° to 70°, with a scanning step size of 0.01°, operated at 40 kV and 40 mA with a Cu X‐ray source (Cu Kα_1,2_, *λ* = 1.54056 Å). The interpretation of the results has been done with the X'Pert Highscore Plus Software (PANalytical, 2004) with PDF‐2 data bank (see Faber & Fawcett, 2002).

### 3.3 DNA extraction and sequencing for metagenomic analysis

The three independent samples obtained from each layer were combined together. Then, 10 g of the combined samples was suspended in 15 mL of pH 7.0 PBS buffer and subjected to three 30‐min cycles in an ultrasonic bath. The cells were collected from the supernatant after each cycle by centrifugation (10 min at 10,000 *g*). Finally, the resulting sediment was incorporated into the first step of the FastDNA SPIN Kit for Soil (MP Biomedicals, Inc.) according to the manufacturer's indications.

The total DNA obtained was fragmented using sonication with a target fragment size of ~400 bp. The DNA fragment distribution was confirmed using a Fragment Analyzer (Advanced Analytical), and libraries were constructed using the Illumina TruSeq DNA kit 2 × 300 bp, following the manufacturer's instructions. The libraries were multiplexed and run on an Illumina MiSeq instrument. Raw data are available from the NCBI's database under BioProject PRJNA803473 (BioSample accessions: SAMN25653288, SAMN25653289; NCBI).

### 3.4 Metagenomic data analysis

Prior to assign taxonomy, the reads were filtered per quality using Trimmomatic 0.39 version (Bolger et al., 2014) with the following parameters: HEADCROP:4 CROP:285 LEADING:35 SLIDINGWINDOW:10:27 MINLEN:75. Then, in order to obtain a taxonomic profile, each sample was uploaded to the Galaxy web platform using the public server of usegalaxy.org (Afgan et al., 2016). There, the taxonomic profile was obtained using the k‐mer approach using the Kraken2 software (Wood & Salzberg, 2014). We used the Silva database (created: 2020‐11‐24TI64216Z, kmer‐len = 35, minimizer‐len = 31, minimizer‐spaces = 6) (Quast et al., 2012). Microbial classification of forward and reverse sequences was performed from their annotations at the lowest taxonomic level by Kraken2. The resulting table with the taxonomic classification was exported in the biom format to analyze it in R using the phyloseq package (McMurdie & Holmes, 2013). The biom file was read in R with the read_biom function from the biomformat package and converted in both a taxonomic table (phyloseq format) using tax table function and OTU (Operational Taxonomic Unit) table function. The phyloseq object created was composed of two samples with 1613 taxa by seven taxonomic ranks.

On the other hand, paired‐end reads were assembled using SPAdes 3.9.0 version (Bankevich et al., 2012) with the ‐‐meta parameter to call the metaSPAdes module. The assemblies were annotated using Prokka 1.11 version (Seemann, 2014), adding the ‐‐metagenome parameter to improve gene prediction.

### 3.5 Metagenomic functional analysis

The functional potential of the microbial communities in the green and orange layers was assessed using HUMAnN2 (Franzosa et al., 2018), which aligns metagenomic reads with the MetaCyc database (Caspi et al., 2014) for metabolic pathway analysis. HUMAnN2 initially normalizes the abundances of gene families and metabolic pathways by the length of the genes to yield RPK (reads per kilobase) values. To enable a robust comparison between the metabolic pathway abundances of the green and orange layers, we further normalized the RPK values to CPM (copies per million). This normalization step is crucial as it accounts for sequencing depth, thus allowing for the comparison of gene abundances between samples with varying sequencing efforts. While CPM normalization does not adjust for gene length, it facilitates an accurate comparison of gene expression levels between samples. The resulting normalized values represent the relative abundance of each gene family or pathway, permitting direct comparisons between samples. Metabolic pathway abundance plots were generated using the ggplot2 package (Wickham, 2016) in R.

### 3.6 Lipid biomarkers extraction and analysis

Lyophilized and ground subsamples (1 g) of the green and orange microbial layer samples were extracted with ultrasound sonication (3 × 15 min) using 15 mL of a 3:1 (v/v) mixture of dichloromethane (DCM) and methanol (MeOH) to obtain a 45 mL of total lipid extract (TLE). Before the extraction, tetracosane‐D50, myristic acid‐D27, and 2‐hexadecanol were added as internal standards. The concentrated and desulfurized TLE (Sánchez‐García et al., 2018) was hydrolyzed overnight with KOH (6% MeOH) at room temperature (Grimalt et al., 1992). Then, a liquid–liquid extraction with *n*‐hexane was performed to recover the neutral fraction and acidification with HCl (2 N) was employed to separate the acidic fraction of the hydrolyzed TLE (details in Sánchez‐García, Carrizo, et al., 2020; Sánchez‐García, Fernández‐Martínez, et al., 2020). Further separation of the neutral fraction into non‐polar (hydrocarbons) and polar (alkanols and sterols) was done according to a method described in detail elsewhere (Sánchez‐García, Fernández‐Martínez, et al., 2020). The acidic fraction was transesterified with BF_3_ in MeOH to produce fatty acid methyl esters (FAMEs), and the polar fraction was trimethylsilylated (N, O‐bis [tri‐methylsilyl] trifluoroacetamide [BSTFA]) to analyze the resulting trimethyl silyl alkanols (details in Sánchez‐García, Carrizo, et al., 2020; Sánchez‐García, Fernández‐Martínez, et al., 2020).

All fractions were analyzed using gas chromatography–mass spectrometry (GC–MS) using 8860 GC System coupled to a 5977B MSD (Agilent Technologies, Santa Clara, CA, USA) operating with electron ionization at 70 eV and scanning from *m*/*z* 50 to 650 (analytical details can be found in Sánchez‐García, Carrizo, et al., 2020; Sánchez‐García, Fernández‐Martínez, et al., 2020). Compound identification was based on retention time and mass spectra comparison with reference materials and the NIST mass spectral database. Quantification was performed with the use of external calibration curves of *n*‐alkanes (C_10_ to C_40_), FAMEs (C_6_ to C_24_) and *n*‐alkanols (C_14_, C_18_, and C_22_), all supplied by Sigma‐Aldrich (Madrid, Spain). Recovery of the internal standards was measured to average 73 ± 15%.

### 3.7 Compound‐specific isotope analysis of lipid biomarkers

Carbon isotopic composition of individual lipid compounds was determined by coupling the gas chromatograph–mass spectrometer (Trace GC 1310 ultra and ISQ QD MS) to the isotope–ratio mass spectrometry system (MAT 253 IRMS, Thermo Fisher Scientific). For the GC analysis, the oven temperature program was set to gradually increase from 70 to 130°C at 20°C min^−1^ and to 300°C at 10°C min^−1^ (held for 15 min), whereas the conditions for IRMS analysis were as follows: electron ionization 100 eV; Faraday cup collector's m/z 44, 45, and 46; and temperature of the CuO/NiO combustion interface at 1000°C. The samples were injected in a PTV injector in splitless mode, with an inlet temperature of 50°C and then a ramp temperature of 2.5°C s^−1^ until 320°C (held 2.5 min), using helium as carrier gas at a constant flow of 1.1 mL min^−1^. The isotopic values of the individual lipids separated by GC were calculated using CO_2_ – spikes of known isotopic composition, introduced directly into the MS source, three times at the beginning and end of every run. Reference mixtures (Indiana University, United States) of known isotopic composition of *n*‐alkanes (A6) and FAMEs (F8) were run every three samples in order to check the accuracy of the isotopic ratio determined by the GC‐IRMS. For the alkanoic acids, the δ13C data were calculated from the FAME values, correcting them for the one carbon atom added in the methanolysis (Abrajano et al., 1994).

## 4 RESULTS

### 4.1 Lake setting

Laguna Verde is located in the northern part of Salar de Antofalla (Figure 1a,b), at the base of an important modern alluvial deposit, which is made up of two fans composed of silt, sand, and gravel (Figure S1). This lake has a “half‐moon” shape and covers an area of approximately 0.2 km^2^. It is geomorphologically composed of numerous depressions in the form of sub‐rounded basins, with depths ranging from 1.5 to 3.5 m (Figure 1c).

During our measurements, Laguna Verde's water exhibited an almost alkaline nature, registering a pH of 8.01 and displaying transparency with light green hues. Our data indicated elevated electrical conductivity values (214.4 μS cm^−1^) and a low dissolved oxygen percentage (6.0%). Surface water temperature measured approximately 11°C in summer and 4°C in winter, based on daytime measurements. At greater depths, the temperature increased to around 15°C, with a thermocline detected 2.5 m below the air–water interface.

Among the ions measured in the water column (Table 1), chloride was the most abundant. Interestingly, chloride, sodium, potassium, and magnesium show a decrease in concentration during winter compared to summer. On the other hand, sulfate and calcium show an increase in concentration during the winter compared to the summer. In addition to this, Table 1 provides measurements of total hardness, chemical oxygen demand (COD), and biological oxygen demand (BOD).

**TABLE 1:** Physicochemical parameters measured in the water column.

Parameters	Summer value	Winter value
Chloride	141.3	122.0
Phosphate	<0.003	<0.003
Nitrate	<0.25	0.67
Nitrite	<0.0002	<0.0002
Ammoniacal nitrogen	<0.0005	<0.0005
Sulfate	5.67	6.49
Calcium	1.61	2.12
Potassium	16.3	1.4
Magnesium	1.68	1.52
Sodium	86.2	72.6
Iron	<0.001	<0.001
Manganese	<0.005	<0.005
C.O.D.5	0.09	<0.005
B.O.D.	1.2	0.7
Total hardness	10.9	11.6

*Note*: All units are expressed in g L^−1^.

### 4.2 Domes morphology and mineralogical composition

#### 4.2.1 Architecture and external morphology

**FIGURE 2:**
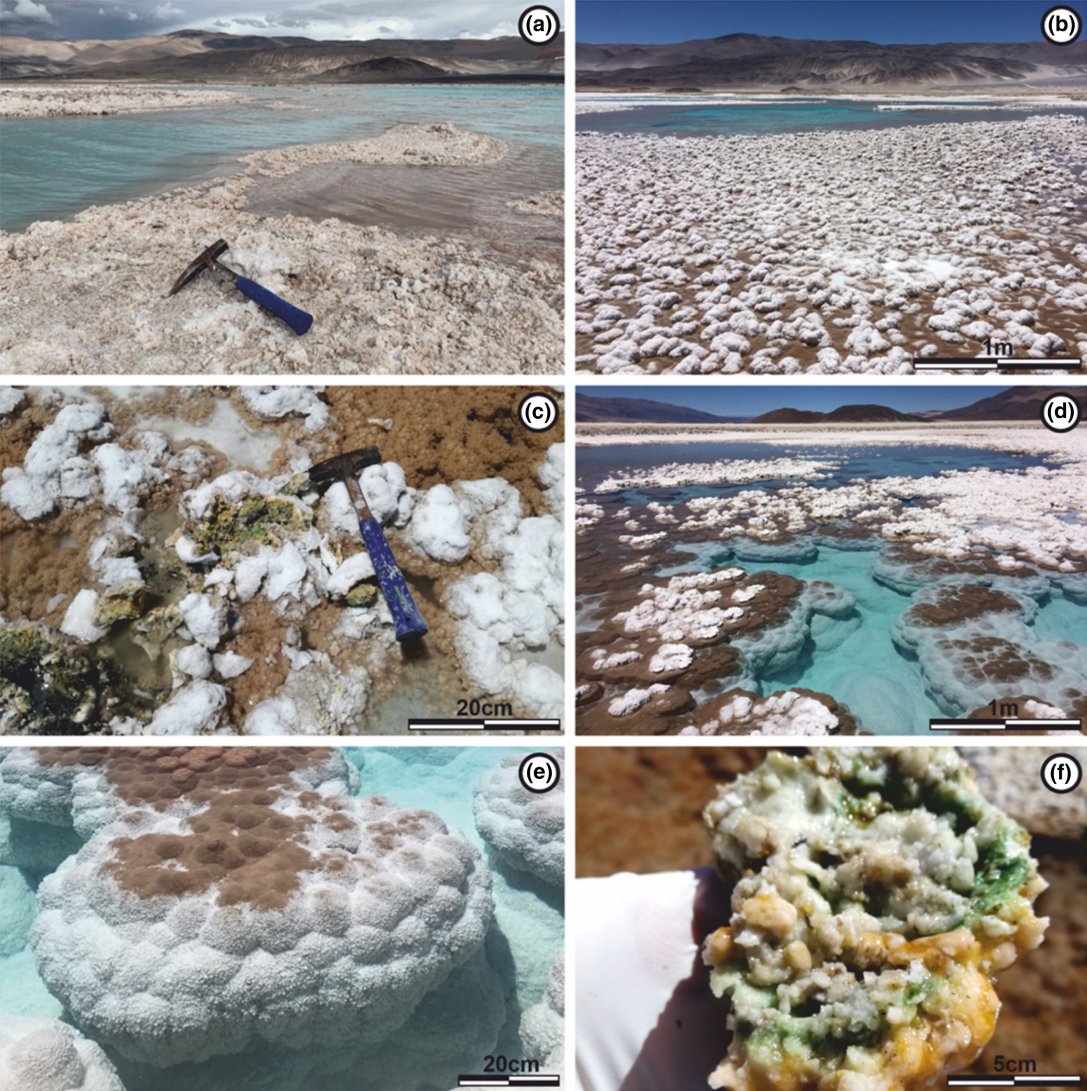
Photographs of the environment of Laguna Verde and the microbial ecosystems present there. (a) Shores of Laguna Verde showing the significant presence of evaporitic minerals precipitated in the surrounding area. (b) Seasonally and periodically emerged and semi‐emerged areas, represented by ephemeral salt flats and evaporitic banks, where salt crusts are present. (c) Salt crusts up to 5 cm thick with an irregular surface, composed of mounds up to 10 cm in diameter. (d) Permanently submerged areas, represented by depressions in the form of sub‐rounded basins. These are populated by evaporitic domic structures, which show a whitish halite cover on top. (e) Flattened domic structures with an external morphology of the botryoidal type composed of lobes up to 20 cm in diameter covering their entire surface. (f) Organic layers (green and orange), observed in the mounds up to 10 cm, used as a model for the study of mineral precipitation.

The surface of Laguna Verde and its surroundings are covered almost entirely by evaporites (Figure 2a). In the shallower areas of the lake, the evaporites appear as crusts up to 5 cm thick. These crusts have an irregular surface, composed of mounds up to 10 cm in diameter, with variable morphology (Figure 2b). When the lake loses depth, these mounds are often exposed to subaerial conditions, resulting in desiccation‐cracked surfaces on top of the mounds (Figure 2c).

In addition to the evaporitic crusts described earlier, there are dome‐shaped structures at the bottom and on the walls of the numerous depressions that make up this lake (Figure 2d). These domes are flattened at the top and have heights of up to 0.5 m and diameters of up to 1.5 m. Their external morphology is botryoidal, consisting of lobes up to 20 cm in diameters that cover their entire surface (Figure 2e), and they show a significant degree of cementation.

Dome‐shaped structures are always found in subaquatic conditions, with the depth of the lake limiting their vertical growth. Lateral growth can occur, with the domes coming into contact with adjacent domes, creating barriers along the lake that extend over significant parts of its surface (Figure 2d).

Both under the evaporitic crusts and inside the domes, the presence of organic matter is observed, positioned close to the air–water interface. In regions exhibiting higher preservation, these organic layers can be organized into two distinct types (Figure 2f), each studied separately from a microbiological standpoint.

#### 4.2.2 Internal morphology

**FIGURE 3:**
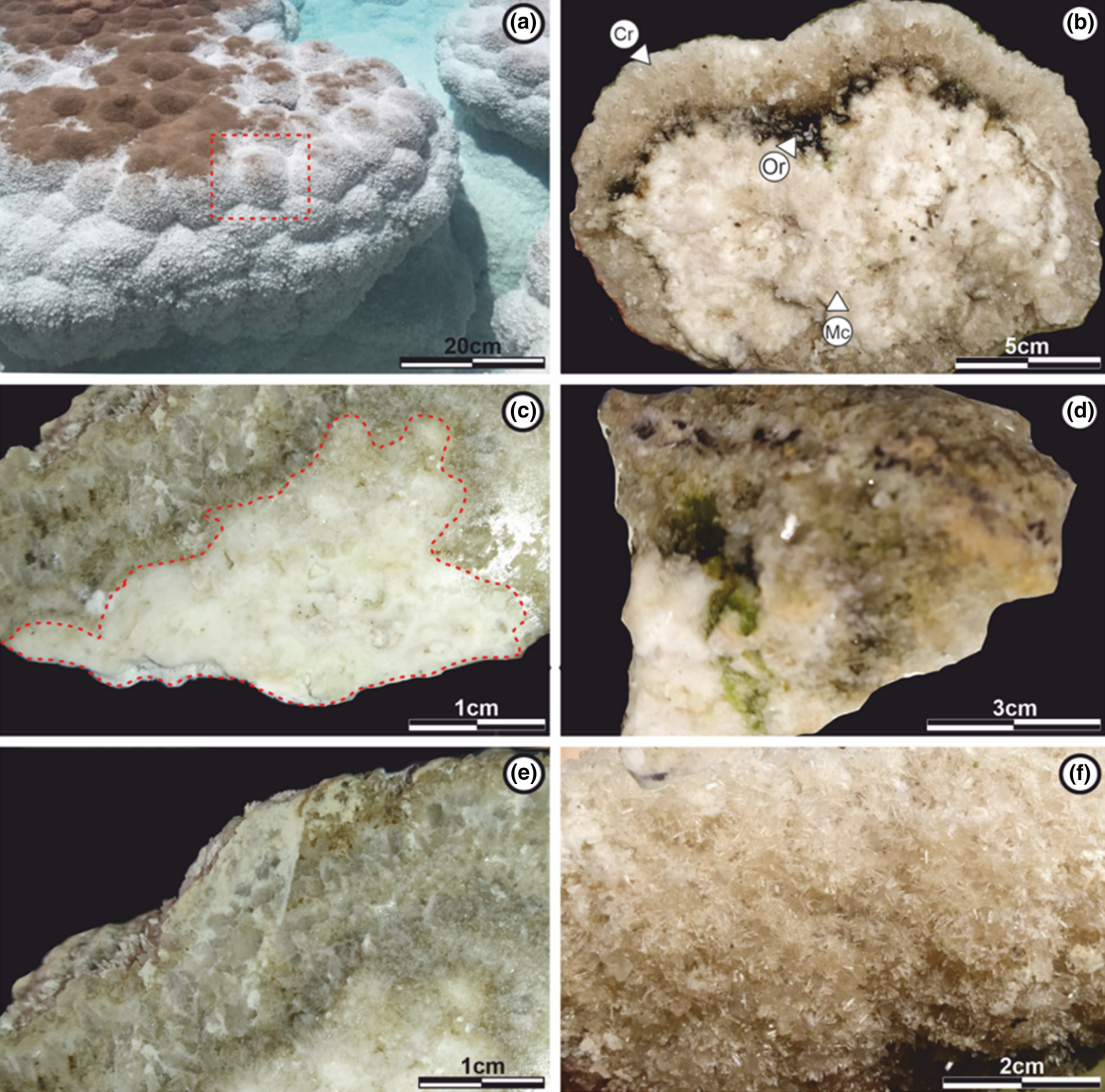
External and internal structure of the evaporite dome‐shaped observed in Laguna Verde. (a) Lobes that form the domes, giving them a botryoidal external aspect. They show a high degree of lithification. (b) Internal structure observed inside the lobes, where we can differentiate three types of mesostructures based on their textural and mineralogical variations. *Organic zone* (Or), *microcrystalline zone* (Mc), and *crystalline zone* (Cr). (c) *Microcrystalline zone* characterized by a whitish microcrystalline texture and a low degree of cementation in contact with the *crystalline zone*. (d) *Organic zone* (greenish color), between *microcrystalline* and *crystalline zone*. (e) *Crystalline zone* formed by layers of gypsum minerals. (f) External surface of the lobes composed of pale brown gypsum crystals. Images (b–f) obtained using a Leica DMS 100 binocular loupe.

Inside, the domes present a heterogeneous and concentric internal structure. Their central part is practically hollow, composed of loose sediment, remains of degrading organic matter, and gas content from putrefaction. Conversely, the lobes forming the domes display the highest degree of lithification. By studying their internal structure, we can differentiate three types of mesostructures based on their textural variations: (i) *microcrystalline zone*, (ii) *organic zone*, and (iii) *crystalline zone* (Figure 3b).

##### Microcrystalline zone

This zone is located in the internal sector of the lobes. It presents a thickness of up to 10 cm and a low degree of cementation. It may be in direct contact with the organic zone or in some cases with the crystalline zone (Figure 3b). At the mesostructural level, the microcrystalline zone shows a homogeneous and whitish texture, where no internal arrangement or euhedral crystal growth can be distinguished (Figure 3c).

**FIGURE 4:**
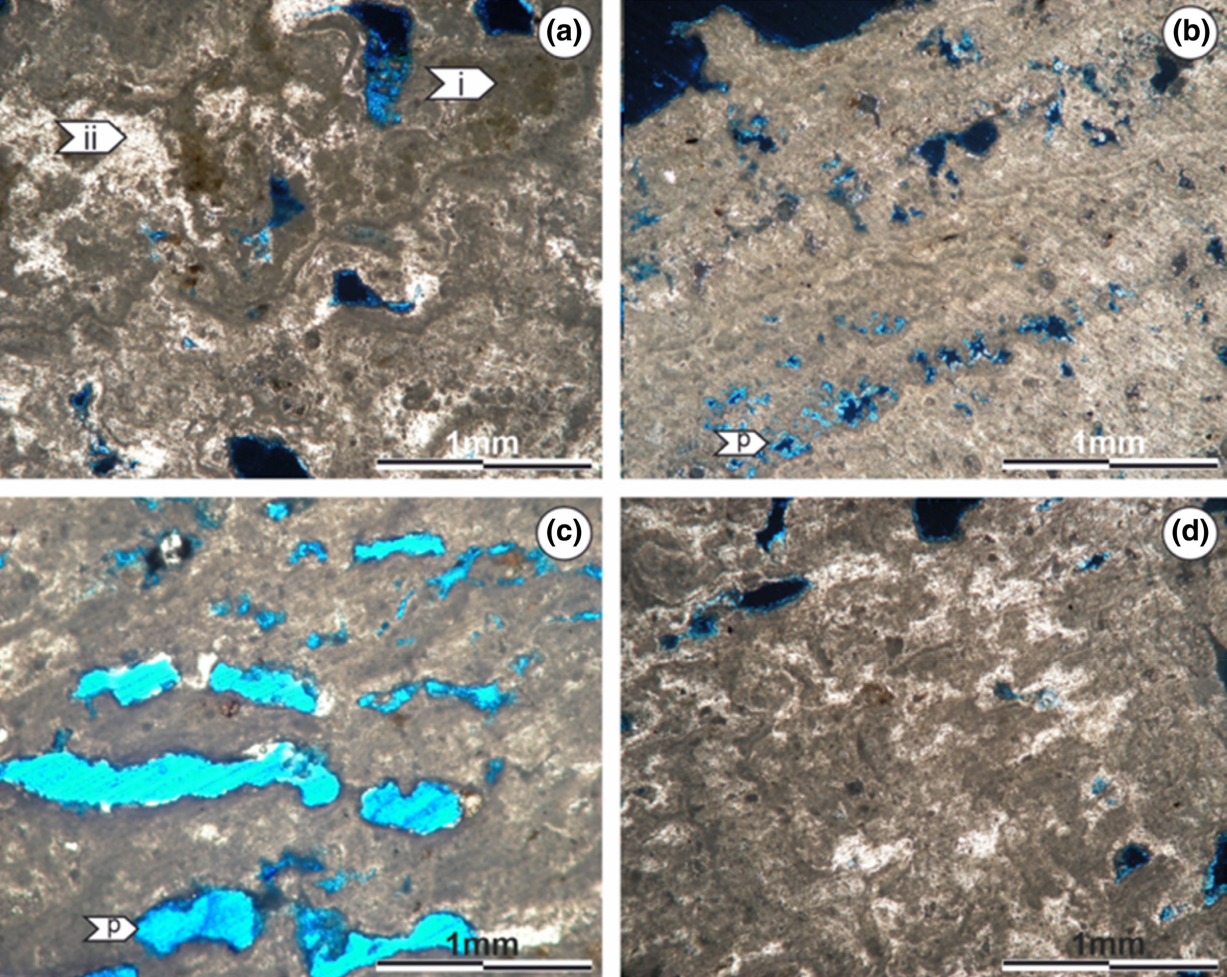
Microstructure of the microcrystalline zone. (a) Alternation between gray (I) and light (II) microcrystalline textures. (b) Presence of lamination (alternating gray and light laminae), with fenestral porosity (P). (c) Important porosity (P), possibly fenestral, developed perpendicular to the growth direction. (d) Sectors where no defined internal structure can be distinguished. However, they continue to alter gray and light microcrystalline textures. Images obtained using a petrographic microscope equipped with a Leica DFC 420 camera.

The total rock X‐ray diffractometry (XRD) indicates that in the microcrystalline zone, gypsum is the predominant mineral, with halite in second place (Figure S2a). Based on the thin sections, it is possible to observe that these minerals are present with dense packed grayish microcrystalline textures or more widely spaced lighter ones (Figure 4a). The microcrystalline zone shows three different microstructural arrangements: I – Laminated sectors, formed by the simple alternation of light and gray laminae (Monty, 1977) (Figure 4b) with good lateral continuity. Lighter laminae reach thicknesses of up to 0.5 mm, while gray laminae do not exceed 0.2 mm. II – Barely laminated sectors, where the laminae have diffuse boundaries and variable thicknesses, making it difficult to distinguish the transition from one to the other. In this sector, gray laminae have a greater prominence than lighter ones (Figure 4c). III – Non‐laminated sectors with a chaotic microstructure, where the alternation between gray and light textures is still observed, without following any pattern (Figure 4a,d).

Throughout the thin sections, different types of porosity are observed (moldic, intraparticulate, caverns, among others) (see Choquette & Pray, 1970). In the laminated and barely laminated sectors, which stand out for their dimensions, the presence of fenestral porosity along the lamination with pores greater than 1 mm in length is observed. It is also important to note the precipitation of pervasive gypsum at the edge of the mesopores or within the micropores (Figure 4c).

##### Organic zone

The organic zone is present in the internal sector of the lobes, above the microcrystalline zone and below the crystalline zone, in direct contact with both (Figure 3b). This zone is identified by its grayish‐brown tonality, thicknesses of up to 5 cm, and a low degree of cementation. The preservation of the organic zone is very varied along the lake. In some domes, a mesostructural arrangement of two orange and green layers can be distinguished in the organic zone (Figure 2f); in others, it is simply absent (not preserved) (Figure 3c), and in certain cases only show a grayish‐brown color (Figure 3d).

**FIGURE 5:**
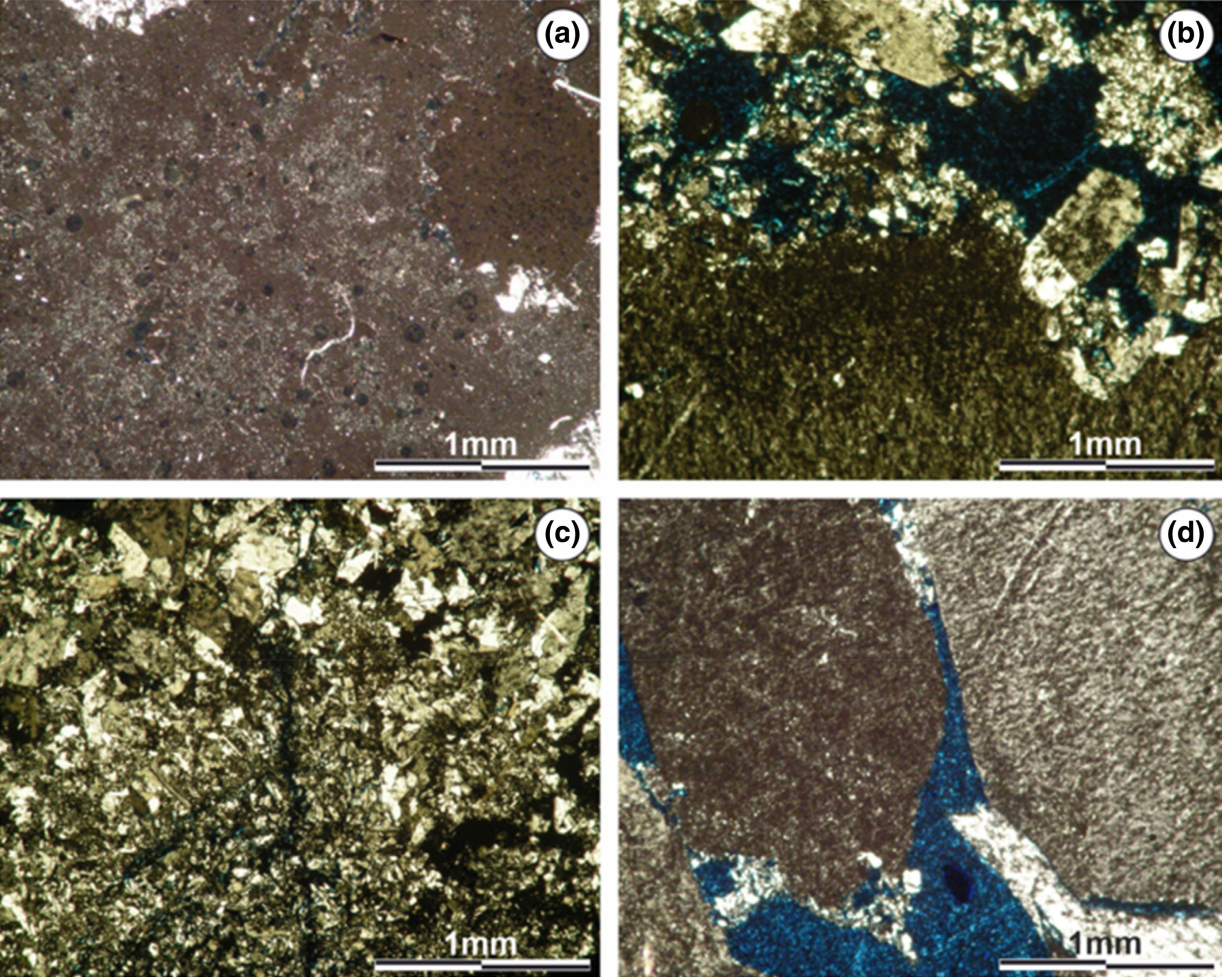
Microstructure of the organic zone. (a) Microcrystalline texture with dense packing and brown to gray colorations. (b) Boundary between the microcrystalline and the crystalline texture. (c) Crystalline texture in the organic zone, where euhedral to subhedral crystals are present. (d) Hexagonal gypsum crystals that have undergone possible dissolution. Images obtained using a petrographic microscope equipped with a Leica DFC 420 camera.

The XRD studies point to gypsum as the most abundant mineral for this zone, followed by halite (Figure S2). The microstructure of the organic zone is heterogeneous, without a defined arrangement. Throughout it, two main textures can be distinguished: (I): microcrystalline facies, densely packed, alternating brown to gray colorations (Figure 5a). (II): Crystalline facies with euhedral to subhedral gypsum crystals showing sizes up to 0.4 mm and without a defined orientation (Figure 5b,c). Several of these crystals exhibit possible dissolution on their edges (Figure 5d).

##### Crystalline zone

The crystalline zone is the outermost of all, covering both the microcrystalline zone and the organic zone. This zone is in direct contact with the lake water and forms the external surface of the domes (Figure 3b). It is up to 8 cm thick, and in some samples, two well‐defined layers with a high degree of cementation can be identified (Figure 3e). The layers are composed of pale brown gypsum crystals (Figure 3f).

**FIGURE 6:**
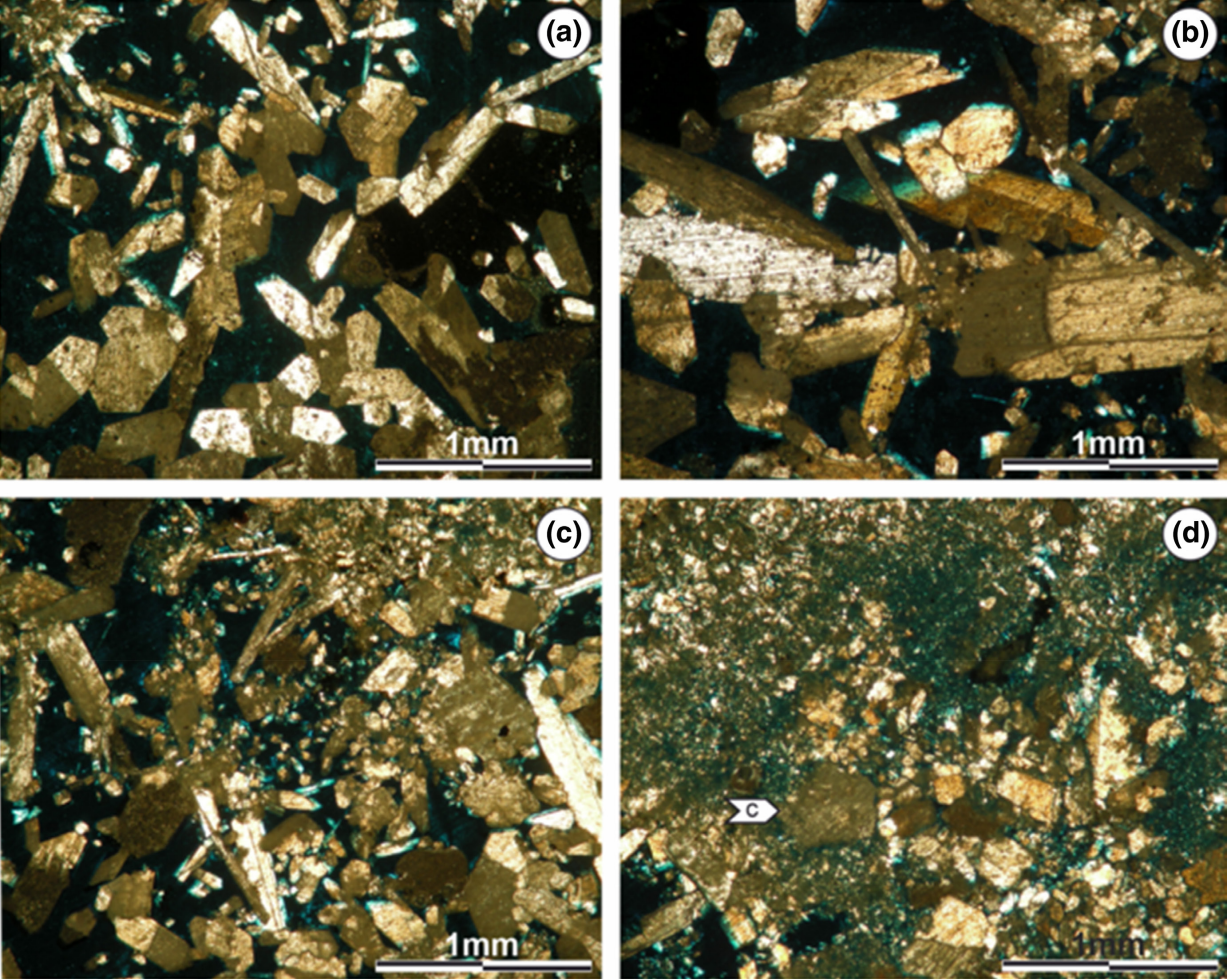
Microstructure of the crystalline zone. (a, b) Euhedral to subhedral fibrous gypsum crystals. (c) Fibrous gypsum crystals alternating with sectors of fine material. (d) Sectors of fine material composed of (c) mineral grains with possible dissolution, close to the organic zone. Images obtained using a petrographic microscope equipped with a Leica DFC 420 camera.

The microstructure of the crystalline zone presents two types of textures: (I): In the outermost zones, euhedral to subhedral fibrous gypsum crystals are observed. These have hexagonal morphology and reach a development of up to 1.5 mm (Figure 6a,b). (II): On the other hand, sectors conformed by fine material are described (Figure 6c,d). In these, the presence of euhedral gypsum fragments (up to 0.2 cm) is highlighted, which appear to show possible dissolution at their edges and are in contact with the organic zone (Figure 6d). As expected, XRD indicates that gypsum continues to be the main mineral (Figure S2).

This zone has a significant porosity, which covers 40% of the thin surface. This porosity is of the intergranular type, and the pores have an important connectivity between them, allowing the passage of water to the center of the domes.

### 4.3 Microbiological analysis

From sequencing by Illumina TruSeq, a total of 114,010,236 raw reads were obtained in the orange layer metagenome and 127,783,895 in the green layer. These raw reads were then filtered by quality, assembled, and annotated. The taxonomic assignment performed with Kraken2 yielded 33,829,422 and 23,632,113 distinct OTUs in the orange and green layers, respectively. Here, each OTU represents a cluster of similar sequence reads that share a certain level of identity − 97% for bacteria and archaea – and can be considered to represent a specific taxonomic rank in the microbial community. The rarefaction curves (Figure S3a) indicate that the sequencing depth achieved for both layers was sufficient to capture the microbial diversity, ensuring that the samples provide a comprehensive representation of the microbial community structure. Subsequently, in order to perform a valid comparison of metabolic pathway abundance between samples, as explained in methodology, the abundances given in RPK were normalized to CPM (copies per million). This normalization takes into account both gene length and sequencing depth.

#### 4.3.1 Alpha‐diversity and taxonomic distribution of metagenomes

The study of the microbial diversity present in the microbial ecosystem of Laguna Verde was carried out through the massive sequencing of metagenomic DNA extracted, independently, from the two layers described previously (Figure 2f). The alpha diversity analysis of the two studied layers revealed that the number of observed species (observed) is higher in the orange layer, but there are no substantial differences between the two layers (Figure S3b). On the other hand, the uncertainty or degree of entropy with respect to the taxonomy of the microbial community (Shannon) and the index related to dominance (Simpson) were both higher in the green layer.

**FIGURE 7:**
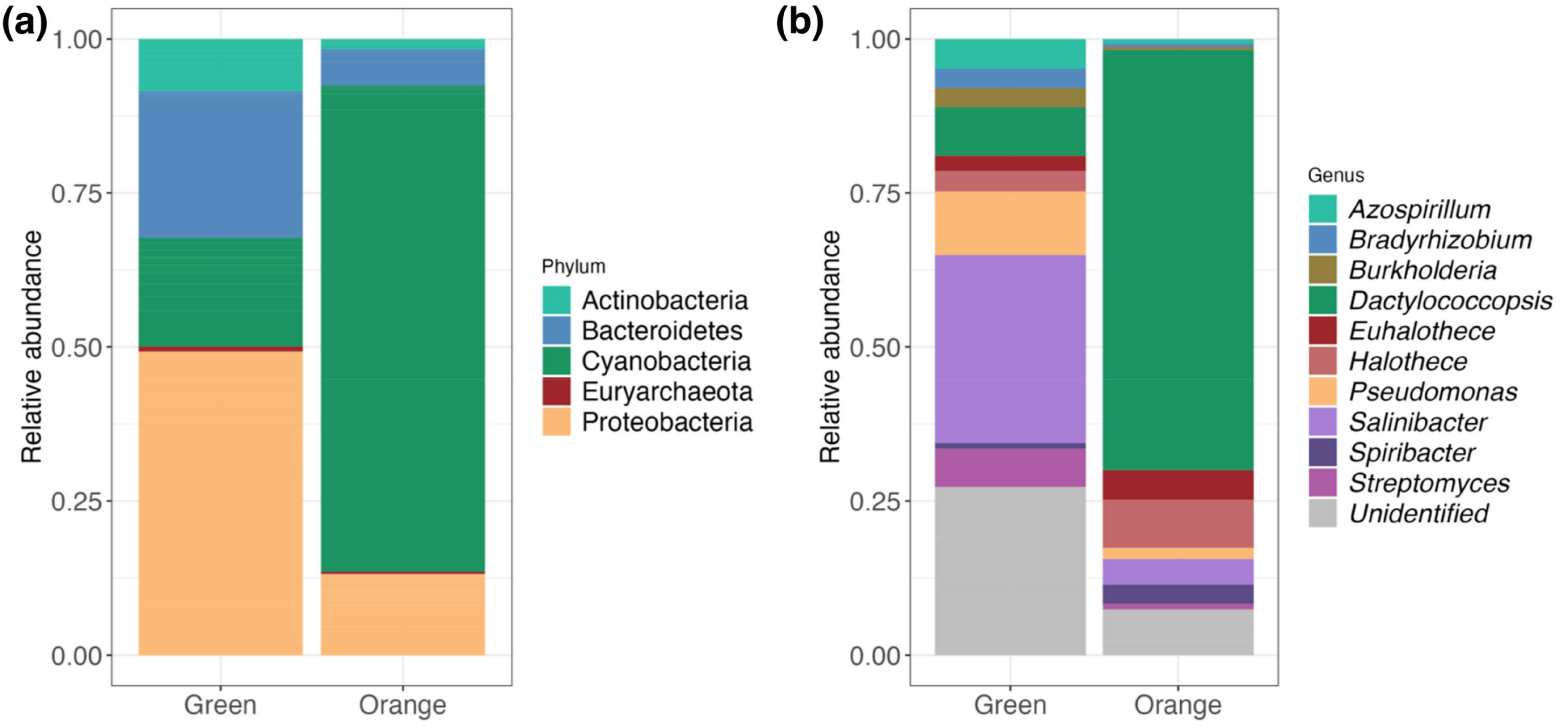
Stacked column graph representing the relative distribution of dominant phyla and genera (a and b, respectively) in the green and orange layer samples. Phyla with an abundance above 0.3% and the 15 most abundant genera have been plotted. Sequences were taxonomically assigned using the Kraken2 database.

Taxonomic analyses of the two layers (green and orange) revealed a high abundance of Cyanobacteria and Proteobacteria (Figure 7) and a minimal presence of archaea. There were remarkable differences between the two layers analyzed, not in terms of the taxa that compose them but in their distribution and abundance. The orange layer is composed of 80% Cyanobacteria, in addition to Proteobacteria (ca. 12.5%) and Bacteroidetes (ca. 6%). The green layer, on the other hand, is mostly composed of Proteobacteria (ca. 50%) and Bacteroidetes (ca. 25%) and to a lesser extent by Cyanobacteria (ca. 16%) and Actinobacteria (ca. 8%) (Figure 7).

The 15 most abundant genera in both layers were plotted (Figure 7). The orange layer, with the highest percentage of Cyanobacteria, is composed of approximately 70% Dactylococcopsis, 8% Halothece, and 5% Euhalothece (Figure 7). On the other hand, the dominant genus in the green layer is Salinibacter (ca. 30%), followed by Pseudomonas (ca. 10%) and Dactylococcopsis (ca. 8%). It is worth noting that, like most Andean microbial ecosystems (AMEs), these samples showed a high percentage of unidentified taxa at the genus level (Figure 7) and also at family, order, and class level (Figure S4).

#### 4.3.2 Metabolic pathways

**FIGURE 8:**
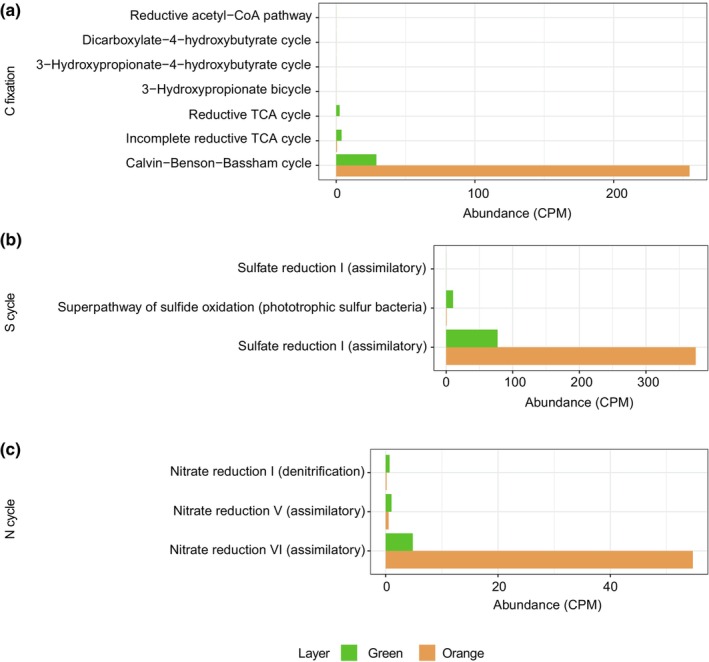
Functional microbial pathways across green and orange layers. Abundance of metabolic pathways associated with mineral precipitation analyzed by HUMANn2 according to the Metacyc database. (a) C fixation pathways. (b) Pathways associated with the S cycle. (c) Pathways associated with the N cycle.

The analysis of the possible metabolic pathways present was performed through HUMAnN 2.0 and using the Metacyc database (Figure 8). The pathways plotted are all those present related to carbon fixation, S cycle, and N cycle (all metabolic pathways detected and their respective abundance in CPM are listed in Table S1). The orange layer was shown to have a much higher abundance of all pathways analyzed compared to the green layer. Regarding C fixation pathways, the main pathway present in both layers is the Calvin–Benson–Bassham photosynthetic fixation pathway (Figure 8). The orange layer shows more than 250 CPM corresponding to this particular pathway, while the green layer has ca. 28 CPM. The reverse Krebs cycle and the incomplete Krebs reverse cycle (TCA reverse and incomplete TCA reverse cycles) are also present, although in low proportions compared to the Calvin cycle, and are more abundant in the green layer. As for the other key C fixing pathways (reducing acetyl‐CoA, dicarboxylate‐4‐hydroxybutyrate, 3‐hydroxypropionate‐4‐hydroxybutyrate, and 3‐hydroxypropionate bicycle), they were not found in either layer.

As for the metabolic pathways associated with the S cycle, the main pathway present is assimilative sulfate reduction (Figure 8), with more than 350 CPM in the orange layer. Strikingly, dissimilatory sulfate reduction was not found in either of the two layers analyzed, which is according to the taxonomic diversity where no sulfate reducing bacteria (SRB) were found (Figure 7). A sulfide oxidation pathway is also present in a lower proportion and mainly in the green layer, associated with phototrophic sulfur bacteria, probably present in purple sulfur bacteria (Gammaproteobacteria) found in both layers (Figure S4).

Finally, the metabolic pathways associated with the N cycle were also evaluated. Nitrate assimilative reduction is the pathway with the highest abundance in the orange layer (ca. 54 CPM), while the dissimilative pathway is present in both layers, but with much lower abundance (Figure 8). The denitrification pathway is also present in low abundance and mostly in the green layer.

### 4.4 Biomarker composition and their compound‐specific carbon isotope analysis (δ^13^C‐CSIA)

The apolar fractions in the green and orange endoevaporitic layer were composed of a series of *n*‐alkanes from C_15_ to C_31_, 1‐alkenes (C_17:1_‐C_22:1_), and isoprenoids (squalene and diploptene) with a general dominance of alkenes with low molecular weights (LMW) (i.e., C_18:1_ and C_19:1_) (Table S2). In the acid fraction, the composition was dominated by mono‐unsaturated C_16:1[ω7]_ and C_18:1[ω9]_ acids, linear (C_16:0_) alkanoic acids, and poly‐unsaturated C_18:2[ω6,9]_, C_20:4[ω3]_ and C_20:5[ω3]_ acids. Terminal branched (*iso*/*anteiso* C_15:0_), cyclopropyl‐acids (Cy_17_ and Cy_19_) and middle‐branched (C_16:0_‐C_18:0_) fatty acids were also found (Table S3). The polar fraction was dominated by phytol, hexadecanol (C_16_), and cholesterol (Table S4).

CSIA (δ^13^C) values for the apolar (hydrocarbon) fraction in the green and orange layer from the endoevaporitic sample were in a range from −32.1‰ to −12.6‰, for alkanoic acids from −29.6‰ to −15.4‰, and for the polar compounds from −30‰ to −16.1‰ (Tables S2–S4). δ^13^C values for principal biomarkers in the apolar fraction from the green and orange layer were as follows: C_17:1_ (−31.6‰ and −32.2‰), C_18:1_ (−28.8‰ and −27.1 ‰), and for heptadecane (−29.3‰, both samples). In the acid fraction, the δ^13^C isotopic composition for the most abundant biomarkers was for mono‐unsaturated C_16:1[ω7]_ (−19.8‰ and −22.1‰), C_18:1[ω9]_ acids (−26.5‰ and −26‰), linear (C_16:0_) from −23.9‰ and −23.8‰, respectively. The δ^13^C values for the poly‐unsaturated C_18:2[ω6,9]_ were −20.8‰ and −29.6‰, for C_20:4[ω3]_ −20‰ and −22.5‰, and for C_20:5[ω3]_ acids from −18.6‰ and −21.4‰. Terminal branched (*iso*/*anteiso* C_15:0_) had a range of values from −18.5‰ and 17.6‰, cyclopropyl‐acid (Cy_17_) with a range of −23.1‰ to −22.7‰ and middle branched (C_16:0_) fatty acids with values from −19.9‰ and −17.5‰. The polar fraction δ^13^C values for the dominated compounds was for phytol −24.6‰, hexadecanol (C_16_) from −19.9‰ and −18.7‰, and cholesterol only measured in the green sample with −16.1‰.

## 5 DISCUSSION

Laguna Verde presents similar physicochemical conditions to other lithifying AMEs with carbonate, gypsum, and halite precipitation (Farías et al., 2020; Vignale et al., 2021). In these systems, seasonal variations are common (Abbott et al., 2003; Castino et al., 2017), affecting the chemical composition of the water (Table 1), the accommodation space of the domes (limited to the permanently submerged areas) (Bergman et al., 2010; Kah et al., 2006; Strohmenger & Jameson, 2017), and possibly the microbial activity (Rouchy & Monty, 2000; Taher, 2014).

The location of Laguna Verde at the edge of Salar de Antofalla, close to alluvial fan deposits, favors the supply of meteoric water to the system. On the other hand, the increase in water temperature at depth can be explained by a source of hydrothermal input at the bottom of the lake. Due to the volcanic activity in the region, hydrothermal input is a common component in several water bodies of the Central Andes (e.g. Albarracín et al., 2016; Della Vedova et al., 2022; Farías et al., 2013; Sancho‐Tomás et al., 2020). The water mixing facilitated by these components positions Laguna Verde as an attractive system for microbialite‐producing microorganisms (Beeler et al., 2020; Chagas et al., 2016; Gomez et al., 2014; Warden et al., 2019).

Dome‐shape structures have been described in lakes of Central Andes, both in microbialitic systems (e.g., Lencina et al., 2021; Villafañe, Cónsole‐Gonella, et al., 2021) and endoevaporitic systems (e.g., Farías et al., 2014; Rasuk et al., 2014; Vignale et al., 2021). Similarly, lobed external surfaces are associated with both types of structures (e.g., Suosaari et al., 2016; Taher, 2014; Vogel et al., 2009; Weston et al., 2019), making it difficult to determine the extent of microbiological influence on them.

### 5.1 Internal structure interpretation

XRD studies show the presence of gypsum, and in minor amounts of halite, throughout the domes. However, their internal structure exhibits significant variations in terms of mesostructure, microstructure, and texture. In hypersaline microbial systems, these variations could reflect environmental conditions such as alkalinity, salinity, bathymetry, and mineral saturation (Chagas et al., 2016; Zeyen et al., 2021). Although microorganisms can influence nucleation processes, the hydrogeochemical evolution of lakes exerts primary control over the texture and mineralogy of the facies that make up these systems (Zeyen et al., 2021).

The microcrystalline textures are interpreted as the primary depositional microstructures in the microcrystalline zone (Reid et al., 2003). Some authors associate the presence of microcrystalline gypsum with meromictic lakes, with a water mixture of different origins, such as those described in Laguna Verde (Bąbel, 2005). The formation of these mineral facies might have been driven by a relative decrease in the amount of Ca^2+^, possibly linked to a higher water balance than that which occurred during the formation of euhedral to subhedral fibrous gypsum crystals in the crystalline zone (Bąbel, 2005; Kobluk & Crawford, 1990). However, some characteristics in the microcrystalline zone, such as lamination and fenestral porosity, suggest that an abiotic genesis may not fully explain these features (Ali‐Bik et al., 2013; Aref & Taj, 2013; Chatalov, 2009; Mata et al., 2012; Sanz‐Montero et al., 2005).

The crystalline zone is characterized by the presence of euhedral to subhedral fibrous gypsum crystals (Reid et al., 2003). As seen in other hypersaline systems, water saturation/supersaturation influences mineral precipitation processes (Babel et al., 2011; Reiss et al., 2021; Taher, 2014). Based on this, the high concentrations of chloride, sodium, potassium, and sulfate in Laguna Verde, added to the crystals, allow us to suggest that gypsum precipitation in the crystalline zone respond to a geochemical process (Reiss et al., 2019, 2021).

In hypersaline environments, evaporation, stratification of the water column, and low circulation during dry seasons create ideal conditions for the supersaturation of brines, leading to the formation of large gypsum crystals (up to 1.5 mm) (Rouchy & Monty, 2000; Strohmenger & Jameson, 2017).

In Central Andes, it has been suggested that gypsum crystals provide protection from desiccation and UV‐B radiation for the endolithic microbial communities thriving in their surroundings, while allowing the passage of photosynthetically active radiation (PAR, 400–700 nm), which is important for the development of photoautotrophs (Farías et al., 2014). However, the extent to which biological activity influences the formation of these crystals is not well‐understood.

It is remarkable that XRD does not detect carbonates in the microcrystalline textures because they are common in mixed‐water environments of the Central Andes (e.g., Albarracín et al., 2015; Della Vedova et al., 2022; Gomez et al., 2014; Villafañe, Lencina, et al., 2021).

Although the concentrations of Ca and Mg in Laguna Verde are lower compared to those in other AMEs (e.g., Villafañe, Lencina, et al., 2021), the lack of detected carbonates does not preclude the potential for these minerals to precipitate at certain stages within the lake's hydrogeochemical cycle. Their absence could be explained by different factors, such as the higher resistance of gypsum with respect to carbonate to acid dissolution (Kobluk & Crawford, 1990) or a pervasive effect of gypsum due to its (post‐depositional) precipitation during fluid circulation through the pore space (Babel et al., 2011; Henares et al., 2016; Laurent et al., 2016; Wang et al., 2022).

Gypsum domes in Central Andes have been classified as either microbialites or endoevaporites (e.g. Farías et al., 2014; Herrero, 2019; Phillips et al., 2023). However, to date, no comprehensive studies have been conducted to identify if the gypsum formation is microbiologically influenced or is a purely chemical process. For this, processes such as gypsum precipitation in the matrix of the extracellular polymeric substance (EPS) (Vogel et al., 2009), precipitation induced by physicochemical conditions generated by metabolic activity (Taher, 2014), or calcium binding to the cell wall of microorganisms providing a nucleation site for gypsum precipitation (Babel, 2004; Cabestrero & Sanz‐Montero, 2018) must be studied in this system.

### 5.2 Diversity and activity of the microbial community in the Laguna Verde AMEs

The microbial community composition within Laguna Verde exhibits significant variability between the green and orange layers. In the orange layer, Cyanobacteria are markedly more abundant, suggesting a dominance in this region (Figure 7). However, in the green layer, despite a substantial presence of Cyanobacteria, there is a higher relative representation of other phyla such as Proteobacteria, Bacteroidetes, and Actinobacteria. This diversity indicates a balanced microbial community with multiple ecological contributors. Moreover, the detection of specific biomarkers for Cyanobacteria in both samples underscores the influence of this group throughout the lake's hydrogeochemical cycle, although not as the sole dominant agent. The detection of diploptene and LMW n‐alkanes (C17) and alkenes (C17:1 and C18:1) was linked to the presence of Cyanobacteria (Carrizo et al., 2022; Grimalt et al., 1991; Rohmer et al., 1984). Other biomarkers detected in the acidic fractions, suggesting the presence of Cyanobacteria, were C16:1[ω7], C18:1[ω9], and C18:1[ω7] (de Oteyza et al., 2011). They are the primary producers in the orange layer, showing that the main metabolic activity is related to obtaining energy through photosynthesis and thus fixing C through this pathway (Figure 8). Although under certain conditions, C fixation correlates directly with carbonate precipitation in organo‐sedimentary systems (Arp et al., 2001, 2010; Bundeleva et al., 2012; Dupraz & Visscher, 2005), in the absence of saturation data, it is not possible to determine whether these processes are related in the ecosystems described here.

The results of metagenomics analyses show that in terms of C fixation pathways described in prokaryotes (Berg et al., 2010), the Laguna Verde microbial system has a high abundance of the Calvin cycle (Figure 8). In this work, we observed that the individual lipids (Tables S2–S4) related to the cyanobacterial community had depleted values (e.g., C17:1 range −31.6‰ to −32.2‰ or the unsaturated acid C18:1ω7/ω9 range −23.0‰ to −27.2‰), which suggest that the reductive pentose phosphate cycle (Calvin–Benson–Bassham cycle) was the relevant biosynthetic pathways for autotrophic growth in the lake. Cyanobacteria using this pathway apply a 13C fractionation between the carbon source (CO2) and their lipids between 10‰ and 22‰ (Sirevåg et al., 1977). This range is within the fractionation values obtained for the cyanobacterial biomarkers (ΔCO2‐lipids: from −23.4‰ to −10.9‰).

Studies in similar systems indicate that photosynthetic CO_2_ uptake by Cyanobacteria induces CaCO_3_ precipitation in hypersaline environments (Aloisi, 2008; Görgen et al., 2020; Kühl et al., 2003; Ludwig, 2004; Taher, 2014).

Regarding the metabolic pathways of S‐cycle, no dissimilatory sulfate reduction was found (Figure 8). This is consistent with the taxonomic distribution, as no SRBs were identified (Figure 7). Although SRBs are not present, our results indicate the presence of the sulfide oxidation pathway (Figure 8), which may be carried out by phototrophic sulfur bacteria (purple sulfur bacteria), mainly belonging to the Gammaproteobacteria, mostly found in the green layer (Figure S4a). Cyanobacteria can also perform anoxygenic photosynthesis by oxidation of sulfide; however, the extent to which this occurs in natural systems is not well‐understood (Klatt et al., 2020). Specific lipid biomarkers such as Cy19:0 have been ascribed with anoxygenic phototrophs (purple sulfur bacteria or PSB and/or green sulfur bacteria or GSB) (Grimalt et al., 1992). In our study, high concentrations of this acid were found on both samples (1.3–4.3 μg g^−1^ dw, orange and green layer, respectively). While sulfide oxidation processes can lead to CaCO_3_ dissolution under aerobic conditions (Dupraz et al., 2009; Görgen et al., 2020), in anaerobiosis, this process can lead to proton uptake and create conditions that favor CaCO_3_ precipitation (Görgen et al., 2020). In addition, the main pathway present associated with the N cycle is assimilatory reduction (VI, MetaCyc database) (Figure 8), which is commonly associated with Cyanobacteria. In these organisms, the function of the nitrate reductase enzyme is linked to ferredoxin as the electron donor (González et al., 2006).

The role of microorganisms in gypsum precipitation is not as well‐understood as it is for carbonate precipitation. However, there have been some studies that suggest that Cyanobacteria may be involved in gypsum precipitation in alkaline aquatic environments. Thompson and Ferris (1990) were the first to describe the involvement of Cyanobacteria (Synechococcus) in gypsum precipitation, and subsequent studies have confirmed the correlation between the presence of Cyanobacteria (determined by the salinity) and the occurrence of gypsum stromatolites (Babel, 2004). In some cases, a thick microbial mat dominated by Cyanobacteria may even limit the growth of gypsum crystals (Babel, 2004). However, further research is needed to fully understand the role of the Cyanobacteria in this ecosystem and its impact on gypsum precipitation.

## 6 CONCLUSIONS

In conclusion, Laguna Verde is a unique water body with favorable conditions for the formation of AMEs. The dome‐shaped structures that populated permanently submerged areas of this lake present a heterogeneous internal structure, composed of a *microcrystalline zone*, an *organic zone*, and a *crystalline zone*. The *organic zone* is dominated by Cyanobacteria, which are involved in photosynthesis and carbon fixation. Although the presence of Cyanobacteria in the *organic zone* suggests their potential role in the formation of the AMEs, more research is needed to fully understand their contribution to the process. Laguna Verde represents a remarkable opportunity to discuss the interactions between microbial communities and evaporitic minerals, providing valuable insights applicable to analogous deposits throughout the history of the Earth.

## AUTHOR CONTRIBUTIONS

**L. A. Saona:** Conceptualization (equal); formal analysis (equal); investigation (equal); methodology (equal); supervision (equal); writing – original draft (equal); writing – review and editing (equal). **P. G. Villafañe:** Conceptualization (equal); formal analysis (equal); investigation (equal); methodology (equal); supervision (equal); writing – original draft (equal); writing – review and editing (equal). **D. Carrizo:** Formal analysis (supporting); investigation (supporting); methodology (supporting); writing – original draft (supporting). **C. Cónsole Gonella:** Conceptualization (equal); funding acquisition (equal); investigation (equal); supervision (equal). **R. F. Néspolo:** Conceptualization (equal); funding acquisition (equal); supervision (equal). **M. E. Farías:** Conceptualization (equal); funding acquisition (equal); investigation (equal); methodology (equal); supervision (equal).

## CONFLICT OF INTEREST STATEMENT

The authors declare no conflicts of interest.

## Supporting information


Data S1

## Data Availability

The metagenomic DNA sequencing data supporting the findings of this study have been deposited in the NCBI's database. These data can be accessed under BioProject PRJNA803473, with the specific BioSample accessions SAMN25653288 and SAMN25653289. The raw sequencing data are available for public access and can be retrieved for further research and analysis.
