# Supplementary material for: Geomicrobiological characterization of the evaporitic ecosystem in the hypersaline lake Laguna Verde (Andean Puna, Northwestern Argentina)
Source: Ecol Evol. 2024 Feb 12;14(2):e10931. doi: 10.1002/ece3.10931 (PMC10859677; doi:10.1002/ece3.10931)
Supplement: Supplementary file 1 — Data S1 [file ECE3-14-e10931-s001.docx]

Supplementary material

Geomicrobiological characterization of the evaporitic ecosystem in the hypersaline lake Laguna Verde (Andean Puna, Northwestern Argentina)


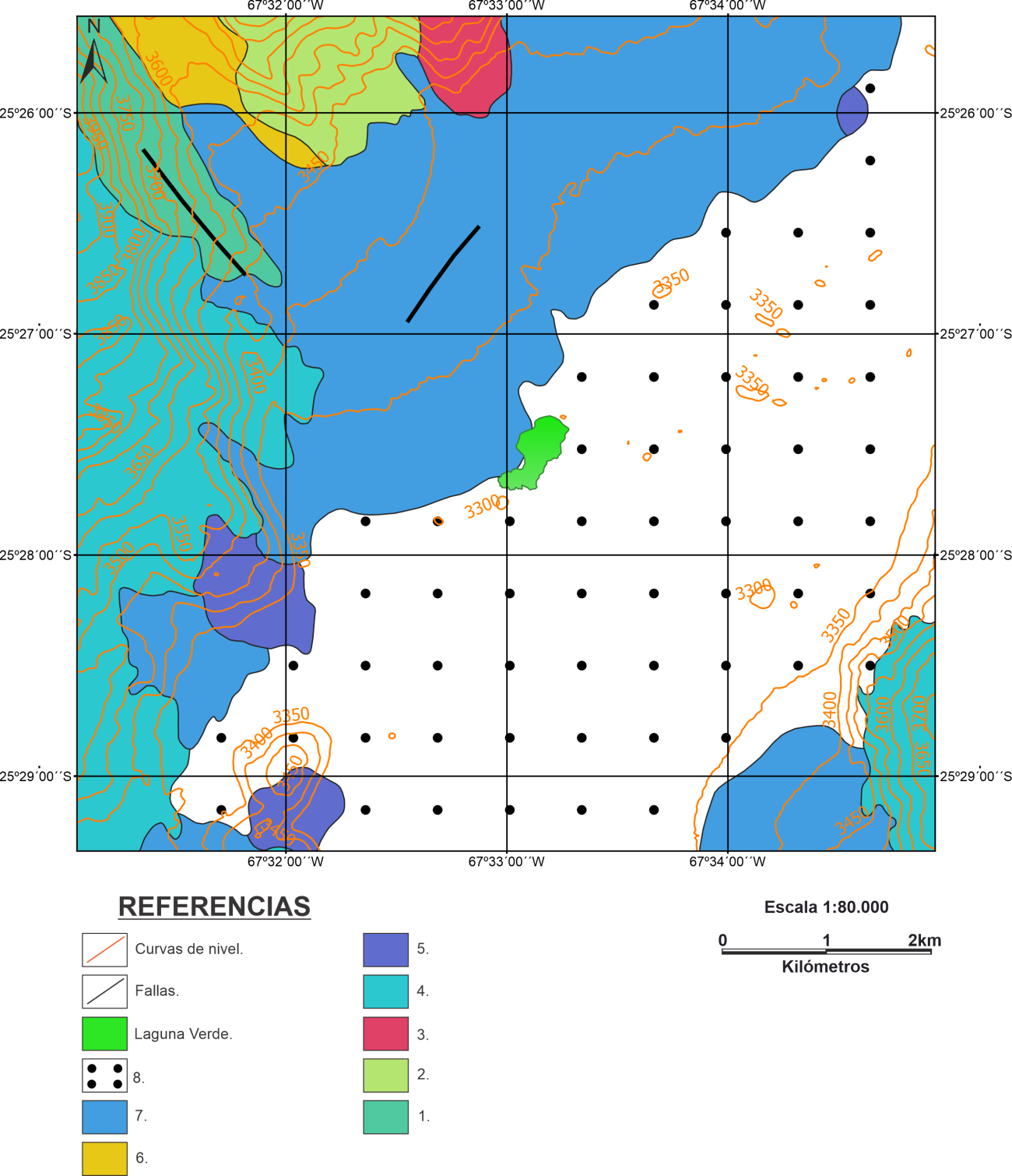


**Fig. S1.-** Geological and stratigraphic framework of Laguna Verde and its surroundings. 1- Vizcachera Formation (Oligocene), 2- Vulcanites (Middle to Upper Miocene), 3- Vulcanites (Upper Miocene), 4- Vulcanites (Lower Pliocene), 5- Basalts (Pleistocene), 6- Fluvial deposits (Holocene), 7- Alluvial deposits (Holocene), 8- Salar (Holocene).


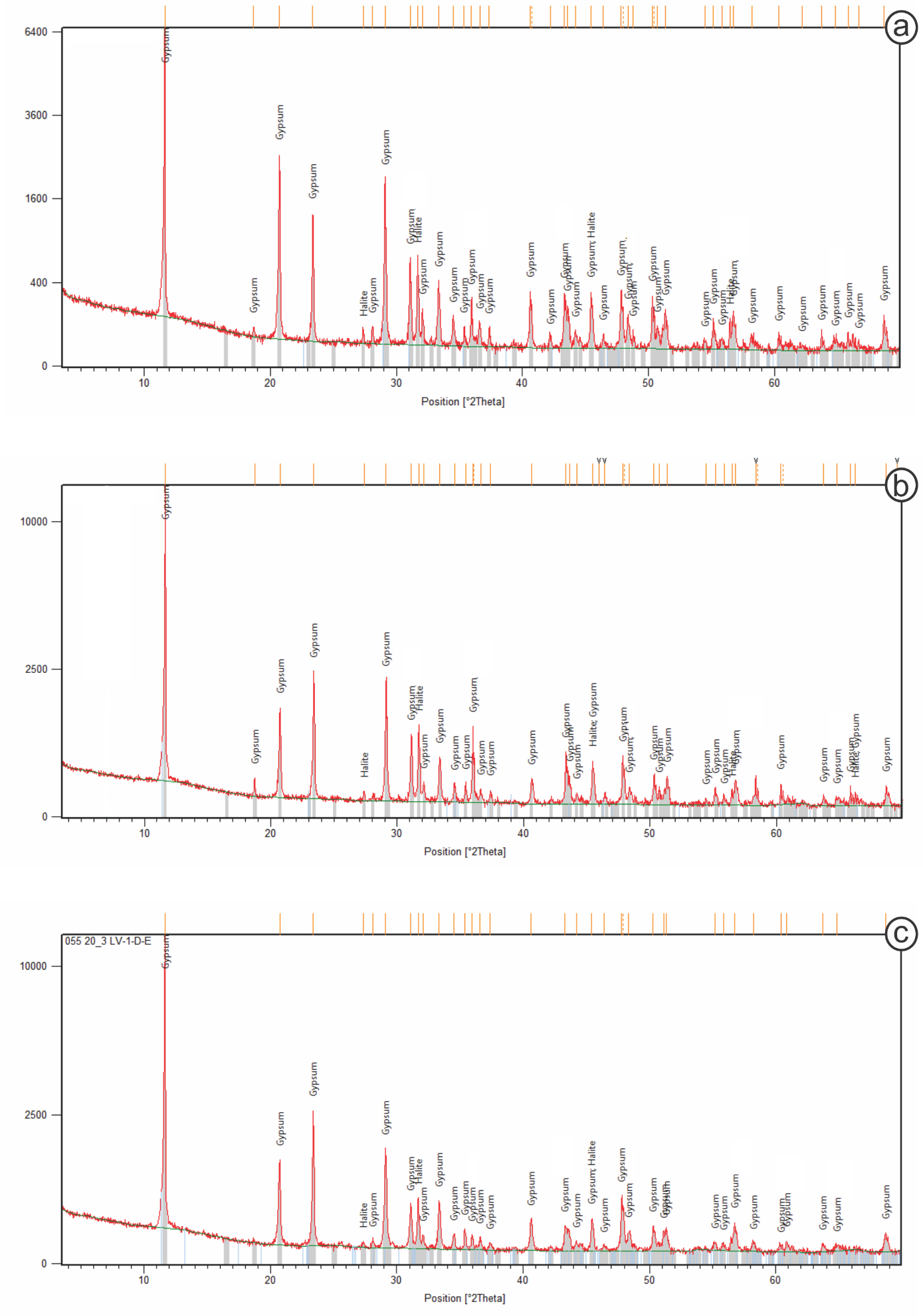


**Fig. S2.-** XRD X-ray diffractometry (XRD) carried out in the microcrystalline zone (a), organic zone (b) and crystalline zone (c).

**Fig. S3.-** a) Rarefaction curve of each set of data (orange and green layers) demonstrating the sequence sample size. b) Observed, Shannon and Simpson alpha diversity measures of orange and green samples.


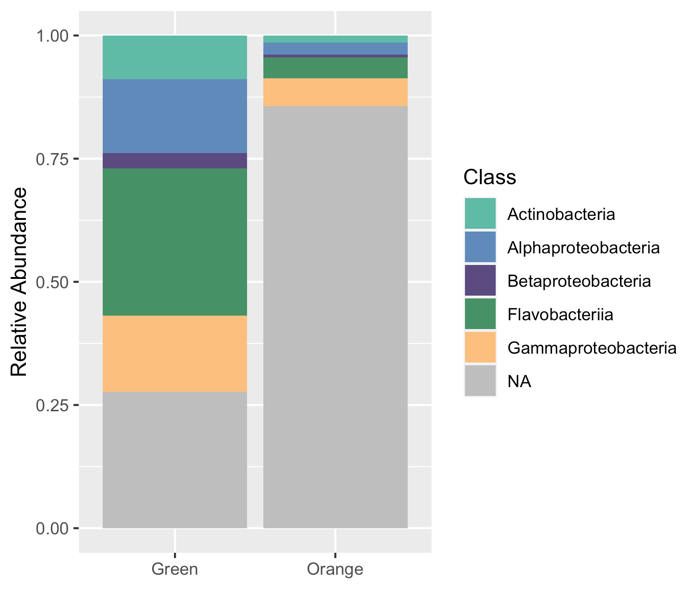

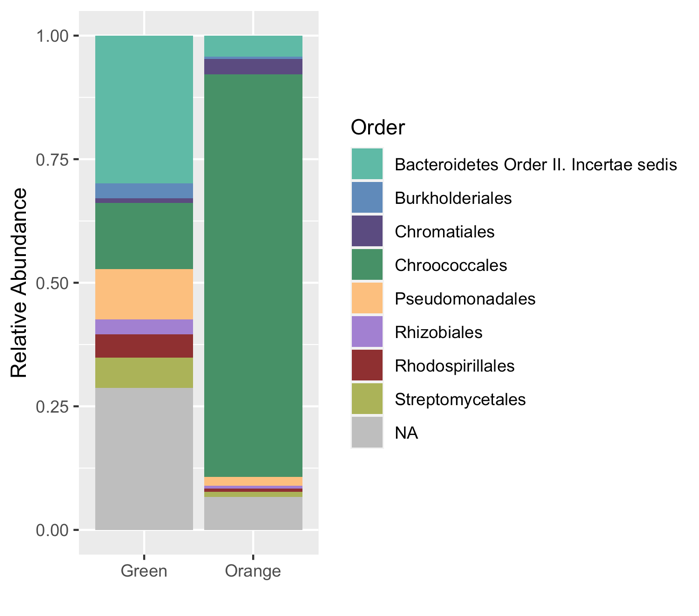

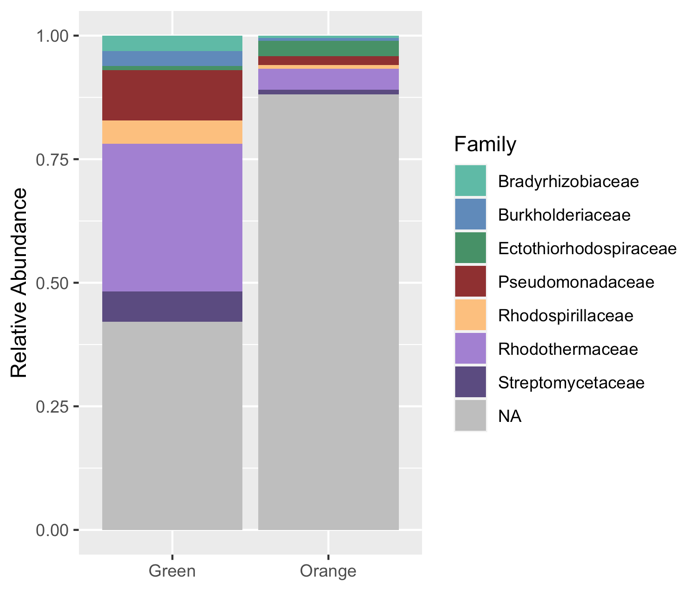


a)

b)

c)

**Fig. S4.-** Stacked column graph representing the relative distribution of dominant Class, Order and Family (a, b and c, respectively) in the green and orange layer samples. The 15 most abundant taxa have been plotted. Sequences were taxonomically assigned using the Kraken2 database.

**Table S1.** Abundance of all metabolic pathway found in both analyzed layers. The abundances are expressed in copies per million (CPM)

| **Pathway** | **Orange Layer (CPM)** | **Green Layer (CPM)** |
| --- | --- | --- |
| 1CMET2-PWY: N10-formyl-tetrahydrofolate biosynthesis | 193.624 | 50.8439 |
| ANAEROFRUCAT-PWY: homolactic fermentation | 61.246 | 30.9805 |
| ANAGLYCOLYSIS-PWY: glycolysis III (from glucose) | 75.7551 | 27.6687 |
| ARGININE-SYN4-PWY: L-ornithine de novo  biosynthesis | 0 | 8.1272 |
| ARGORNPROST-PWY: arginine, ornithine and proline interconversion | 0 | 1.68797 |
| ARGSYN-PWY: L-arginine biosynthesis I (via L-ornithine) | 201.433 | 23.4321 |
| ARGSYNBSUB-PWY: L-arginine biosynthesis II (acetyl cycle) | 178.588 | 19.1021 |
| ARO-PWY: chorismate biosynthesis I | 226.61 | 22.7757 |
| ASPASN-PWY: superpathway of L-aspartate and L-asparagine biosynthesis | 1.31869 | 5.41856 |
| BIOTIN-BIOSYNTHESIS-PWY: biotin biosynthesis I | 6.13127 | 1.78761 |
| BRANCHED-CHAIN-AA-SYN-PWY: superpathway of branched amino acid biosynthesis | 128.367 | 60.055 |
| CALVIN-PWY: Calvin-Benson-Bassham cycle | 254.707 | 28.9466 |
| CENTFERM-PWY: pyruvate fermentation to butanoate | 0 | 0.239939 |
| CHLOROPHYLL-SYN: chlorophyllide a biosynthesis I (aerobic, light-dependent) | 5.9137 | 3.16472 |
| CITRULBIO-PWY: L-citrulline biosynthesis | 0 | 10.2237 |
| COA-PWY-1: coenzyme A biosynthesis II (mammalian) | 148.224 | 21.8785 |
| COA-PWY: coenzyme A biosynthesis I | 196.823 | 33.8652 |
| COBALSYN-PWY: adenosylcobalamin salvage from cobinamide I | 0 | 0.447716 |
| COLANSYN-PWY: colanic acid building blocks biosynthesis | 2.65052 | 4.84172 |
| COMPLETE-ARO-PWY: superpathway of aromatic amino acid biosynthesis | 237.514 | 25.2278 |
| CRNFORCAT-PWY: creatinine degradation I | 0.242305 | 0.333742 |
| DAPLYSINESYN-PWY: L-lysine biosynthesis I | 56.249 | 24.073 |
| DENITRIFICATION-PWY: nitrate reduction I (denitrification) | 0.164187 | 0.70555 |
| DENOVOPURINE2-PWY: superpathway of purine nucleotides de novo biosynthesis II | 14.2606 | 38.6083 |
| DTDPRHAMSYN-PWY: dTDP-L-rhamnose biosynthesis I | 218.484 | 37.2276 |
| FAO-PWY: fatty acid &beta;-oxidation I | 0 | 3.41353 |
| FASYN-ELONG-PWY: fatty acid elongation -- saturated | 5.75305 | 1.37085 |
| FASYN-INITIAL-PWY: superpathway of fatty acid biosynthesis initiation (E. coli) | 4.05174 | 0.858694 |
| FERMENTATION-PWY: mixed acid fermentation | 2.0022 | 7.27697 |
| GALACTUROCAT-PWY: D-galacturonate degradation I | 0.165078 | 0.250791 |
| GLUCONEO-PWY: gluconeogenesis I | 16.5454 | 13.5939 |
| GLUTORN-PWY: L-ornithine biosynthesis | 152.225 | 22.9739 |
| GLYCOGENSYNTH-PWY: glycogen biosynthesis I (from ADP-D-Glucose) | 2.07289 | 5.15245 |
| GLYCOLYSIS-E-D: superpathway of glycolysis and Entner-Doudoroff | 5.03136 | 3.21124 |
| GLYCOLYSIS-TCA-GLYOX-BYPASS: superpathway of glycolysis, pyruvate dehydrogenase, TCA, and glyoxylate bypass | 23.9161 | 15.6237 |
| GLYCOLYSIS: glycolysis I (from glucose 6-phosphate) | 64.1641 | 38.6896 |
| GLYOXYLATE-BYPASS: glyoxylate cycle | 20.5219 | 11.3276 |
| HEME-BIOSYNTHESIS-II: heme biosynthesis I (aerobic) | 321.917 | 45.5984 |
| HISDEG-PWY: L-histidine degradation I | 0.289964 | 3.09912 |
| HISTSYN-PWY: L-histidine biosynthesis | 248.337 | 41.0459 |
| HOMOSER-METSYN-PWY: L-methionine biosynthesis I | 25.5351 | 18.8756 |
| HSERMETANA-PWY: L-methionine biosynthesis III | 109.362 | 49.3123 |
| ILEUSYN-PWY: L-isoleucine biosynthesis I (from threonine) | 327.868 | 70.5755 |
| MET-SAM-PWY: superpathway of S-adenosyl-L-methionine biosynthesis | 47.6105 | 28.2112 |
| METH-ACETATE-PWY: methanogenesis from acetate | 0 | 3.90828 |
| METSYN-PWY: L-homoserine and L-methionine biosynthesis | 41.8791 | 25.4785 |
| NAGLIPASYN-PWY: lipid IVA biosynthesis | 9.95246 | 7.35879 |
| NONOXIPENT-PWY: pentose phosphate pathway (non-oxidative branch) | 248.835 | 58.7949 |
| OANTIGEN-PWY: O-antigen building blocks biosynthesis (E. coli) | 197.019 | 20.825 |
| P101-PWY: ectoine biosynthesis | 31.3469 | 23.03 |
| P105-PWY: TCA cycle IV (2-oxoglutarate decarboxylase) | 29.1693 | 17.7343 |
| P108-PWY: pyruvate fermentation to propanoate I | 0.788545 | 5.63958 |
| P125-PWY: superpathway of (R,R)-butanediol biosynthesis | 2.44496 | 0.456545 |
| P161-PWY: acetylene degradation | 0 | 0.233023 |
| P164-PWY: purine nucleobases degradation I (anaerobic) | 0.316109 | 0.650762 |
| P185-PWY: formaldehyde assimilation III (dihydroxyacetone cycle) | 0.658111 | 0.380048 |
| P221-PWY: octane oxidation | 1.39672 | 0.688211 |
| P23-PWY: reductive TCA cycle I | 0 | 2.43749 |
| P4-PWY: superpathway of L-lysine, L-threonine and L-methionine biosynthesis I | 26.0766 | 10.7397 |
| P42-PWY: incomplete reductive TCA cycle | 0.587785 | 3.83681 |
| PANTO-PWY: phosphopantothenate biosynthesis I | 111.86 | 10.4168 |
| PANTOSYN-PWY: pantothenate and coenzyme A biosynthesis I | 139.409 | 16.1708 |
| PENTOSE-P-PWY: pentose phosphate pathway | 3.81644 | 2.38322 |
| PEPTIDOGLYCANSYN-PWY: peptidoglycan biosynthesis I (meso-diaminopimelate containing) | 217.992 | 19.5807 |
| PHOSLIPSYN-PWY: superpathway of phospholipid biosynthesis I (bacteria) | 28.5527 | 16.066 |
| PHOTOALL-PWY: oxygenic photosynthesis | 266.882 | 27.6138 |
| POLYISOPRENSYN-PWY: polyisoprenoid biosynthesis (E. coli) | 10.4867 | 4.63863 |
| PPGPPMET-PWY: ppGpp biosynthesis | 7.61696 | 17.4477 |
| PROTOCATECHUATE-ORTHO-CLEAVAGE-PWY: protocatechuate degradation II (ortho-cleavage pathway) | 0.153339 | 0 |
| PRPP-PWY: superpathway of histidine, purine, and pyrimidine biosynthesis | 0 | 0.874956 |
| PWY-101: photosynthesis light reactions | 455.318 | 52.6272 |
| PWY-1042: glycolysis IV (plant cytosol) | 191.068 | 54.0754 |
| PWY-1269: CMP-3-deoxy-D-manno-octulosonate biosynthesis I | 5.32664 | 4.94919 |
| PWY-241: C4 photosynthetic carbon assimilation cycle, NADP-ME type | 19.5359 | 20.4009 |
| PWY-2941: L-lysine biosynthesis II | 14.77 | 4.29696 |
| PWY-2942: L-lysine biosynthesis III | 124.769 | 32.4918 |
| PWY-3001: superpathway of L-isoleucine biosynthesis I | 128.173 | 38.9051 |
| PWY-3781: aerobic respiration I (cytochrome c) | 335.125 | 112.907 |
| PWY-3801: sucrose degradation II (sucrose synthase) | 3.20093 | 0 |
| PWY-3841: folate transformations II | 217.925 | 78.0086 |
| PWY-4041: &gamma;-glutamyl cycle | 0.916109 | 5.91449 |
| PWY-4242: pantothenate and coenzyme A biosynthesis III | 119.291 | 12.6902 |
| PWY-4722: creatinine degradation II | 0 | 1.82372 |
| PWY-4981: L-proline biosynthesis II (from arginine) | 62.6985 | 15.5735 |
| PWY-4984: urea cycle | 0 | 0.745366 |
| PWY-5028: L-histidine degradation II | 0 | 0.194662 |
| PWY-5083: NAD/NADH phosphorylation and dephosphorylation | 11.3717 | 8.88565 |
| PWY-5097: L-lysine biosynthesis VI | 261.856 | 26.5316 |
| PWY-5100: pyruvate fermentation to acetate and lactate II | 5.4397 | 6.24147 |
| PWY-5101: L-isoleucine biosynthesis II | 0 | 6.44218 |
| PWY-5103: L-isoleucine biosynthesis III | 109.011 | 60.055 |
| PWY-5104: L-isoleucine biosynthesis IV | 7.51663 | 16.0573 |
| PWY-5136: fatty acid &beta;-oxidation II (peroxisome) | 0 | 2.99395 |
| PWY-5138: unsaturated, even numbered fatty acid &beta;-oxidation | 1.06834 | 2.23789 |
| PWY-5154: L-arginine biosynthesis III (via N-acetyl-L-citrulline) | 26.7365 | 20.3916 |
| PWY-5173: superpathway of acetyl-CoA biosynthesis | 59.2626 | 22.5328 |
| PWY-5177: glutaryl-CoA degradation | 0 | 2.27604 |
| PWY-5188: tetrapyrrole biosynthesis I (from glutamate) | 234.245 | 31.9904 |
| PWY-5189: tetrapyrrole biosynthesis II (from glycine) | 5.4833 | 6.68145 |
| PWY-5198: factor 420 biosynthesis | 0.464613 | 0 |
| PWY-5304: superpathway of sulfur oxidation (Acidianus ambivalens) | 2.33052 | 0 |
| PWY-5345: superpathway of L-methionine biosynthesis (by sulfhydrylation) | 53.3757 | 44.2751 |
| PWY-5347: superpathway of L-methionine biosynthesis (transsulfuration) | 22.5302 | 13.5792 |
| PWY-5464: superpathway of cytosolic glycolysis (plants), pyruvate dehydrogenase and TCA cycle | 56.8714 | 32.0114 |
| PWY-5484: glycolysis II (from fructose 6-phosphate) | 41.0868 | 32.7231 |
| PWY-5505: L-glutamate and L-glutamine biosynthesis | 9.9972 | 11.1831 |
| PWY-5531: chlorophyllide a biosynthesis II (anaerobic) | 4.96783 | 3.29393 |
| PWY-561: superpathway of glyoxylate cycle and fatty acid degradation | 0 | 5.39895 |
| PWY-5659: GDP-mannose biosynthesis | 18.273 | 4.24742 |
| PWY-5667: CDP-diacylglycerol biosynthesis I | 198.046 | 18.2359 |
| PWY-5675: nitrate reduction V (assimilatory) | 0.527359 | 1.03771 |
| PWY-5686: UMP biosynthesis | 248.634 | 48.1199 |
| PWY-5690: TCA cycle II (plants and fungi) | 40.7068 | 40.045 |
| PWY-5695: urate biosynthesis/inosine 5'-phosphate degradation | 94.2086 | 40.3684 |
| PWY-5723: Rubisco shunt | 268.256 | 53.4424 |
| PWY-5747: 2-methylcitrate cycle II | 5.05587 | 7.14553 |
| PWY-5791: 1,4-dihydroxy-2-naphthoate biosynthesis II (plants) | 2.97595 | 0 |
| PWY-5837: 1,4-dihydroxy-2-naphthoate biosynthesis I | 2.97595 | 0 |
| PWY-5838: superpathway of menaquinol-8 biosynthesis I | 7.22553 | 0 |
| PWY-5840: superpathway of menaquinol-7 biosynthesis | 0.702255 | 0 |
| PWY-5855: ubiquinol-7 biosynthesis (prokaryotic) | 11.9164 | 18.9599 |
| PWY-5856: ubiquinol-9 biosynthesis (prokaryotic) | 11.9164 | 18.9599 |
| PWY-5857: ubiquinol-10 biosynthesis (prokaryotic) | 11.9164 | 18.9599 |
| PWY-5861: superpathway of demethylmenaquinol-8 biosynthesis | 4.9782 | 0 |
| PWY-5863: superpathway of phylloquinol biosynthesis | 3.22177 | 0 |
| PWY-5897: superpathway of menaquinol-11 biosynthesis | 6.80529 | 0 |
| PWY-5898: superpathway of menaquinol-12 biosynthesis | 6.80529 | 0 |
| PWY-5899: superpathway of menaquinol-13 biosynthesis | 6.80529 | 0 |
| PWY-5913: TCA cycle VI (obligate autotrophs) | 40.7864 | 36.4123 |
| PWY-5918: superpathay of heme biosynthesis from glutamate | 247.004 | 25.875 |
| PWY-5920: superpathway of heme biosynthesis from glycine | 10.7583 | 10.6981 |
| PWY-5973: cis-vaccenate biosynthesis | 29.5065 | 28.273 |
| PWY-5989: stearate biosynthesis II (bacteria and plants) | 4.33352 | 1.03845 |
| PWY-6121: 5-aminoimidazole ribonucleotide biosynthesis I | 229.696 | 43.4969 |
| PWY-6122: 5-aminoimidazole ribonucleotide biosynthesis II | 269.517 | 47.9361 |
| PWY-6123: inosine-5'-phosphate biosynthesis I | 203.921 | 30.5419 |
| PWY-6124: inosine-5'-phosphate biosynthesis II | 202.881 | 24.8206 |
| PWY-6125: superpathway of guanosine nucleotides de novo biosynthesis II | 183.292 | 71.9316 |
| PWY-6126: superpathway of adenosine nucleotides de novo biosynthesis II | 105.517 | 68.3669 |
| PWY-6147: 6-hydroxymethyl-dihydropterin diphosphate biosynthesis I | 8.26332 | 3.40769 |
| PWY-6151: S-adenosyl-L-methionine cycle I | 0 | 0.577758 |
| PWY-6163: chorismate biosynthesis from 3-dehydroquinate | 211.033 | 20.077 |
| PWY-6168: flavin biosynthesis III (fungi) | 49.3802 | 28.1797 |
| PWY-6174: mevalonate pathway II (archaea) | 3.23438 | 1.30312 |
| PWY-6277: superpathway of 5-aminoimidazole ribonucleotide biosynthesis | 269.517 | 47.9361 |
| PWY-6282: palmitoleate biosynthesis I (from (5Z)-dodec-5-enoate) | 4.49702 | 1.03744 |
| PWY-6305: putrescine biosynthesis IV | 133.157 | 11.2307 |
| PWY-6317: galactose degradation I (Leloir pathway) | 0.513596 | 0.273694 |
| PWY-6318: L-phenylalanine degradation IV (mammalian, via side chain) | 6.99765 | 9.13846 |
| PWY-6349: CDP-archaeol biosynthesis | 1.23283 | 0.628082 |
| PWY-6353: purine nucleotides degradation II (aerobic) | 0.729641 | 2.68898 |
| PWY-6385: peptidoglycan biosynthesis III (mycobacteria) | 215.434 | 19.4723 |
| PWY-6386: UDP-N-acetylmuramoyl-pentapeptide biosynthesis II (lysine-containing) | 186.441 | 16.9052 |
| PWY-6387: UDP-N-acetylmuramoyl-pentapeptide biosynthesis I (meso-diaminopimelate containing) | 216.429 | 19.9471 |
| PWY-6396: superpathway of 2,3-butanediol biosynthesis | 0 | 0.771898 |
| PWY-6507: 4-deoxy-L-threo-hex-4-enopyranuronate degradation | 0.591347 | 0.79232 |
| PWY-6519: 8-amino-7-oxononanoate biosynthesis I | 5.0829 | 1.45798 |
| PWY-6527: stachyose degradation | 0.264392 | 0.226849 |
| PWY-6545: pyrimidine deoxyribonucleotides de novo biosynthesis III | 4.24932 | 26.167 |
| PWY-6549: L-glutamine biosynthesis III | 30.2256 | 27.6129 |
| PWY-6588: pyruvate fermentation to acetone | 0.339361 | 2.30504 |
| PWY-6590: superpathway of Clostridium acetobutylicum acidogenic fermentation | 0 | 0.305034 |
| PWY-6606: guanosine nucleotides degradation II | 0 | 1.03128 |
| PWY-6608: guanosine nucleotides degradation III | 0.399072 | 2.32578 |
| PWY-6609: adenine and adenosine salvage III | 83.3844 | 30.4888 |
| PWY-6628: superpathway of L-phenylalanine biosynthesis | 0 | 0.930551 |
| PWY-6676: superpathway of sulfide oxidation (phototrophic sulfur bacteria) | 0.711561 | 10.3194 |
| PWY-6700: queuosine biosynthesis | 311.021 | 73.3816 |
| PWY-6703: preQ0 biosynthesis | 264.879 | 29.5895 |
| PWY-6708: ubiquinol-8 biosynthesis (prokaryotic) | 11.9164 | 18.9599 |
| PWY-6737: starch degradation V | 0.76165 | 0.742442 |
| PWY-6749: CMP-legionaminate biosynthesis I | 0.202497 | 0.100902 |
| PWY-6785: hydrogen production VIII | 4.98535 | 18.151 |
| PWY-6859: all-trans-farnesol biosynthesis | 6.32611 | 2.88084 |
| PWY-6876: isopropanol biosynthesis | 0.339361 | 2.30504 |
| PWY-6891: thiazole biosynthesis II (Bacillus) | 3.83481 | 1.60151 |
| PWY-6892: thiazole biosynthesis I (E. coli) | 4.77918 | 6.94704 |
| PWY-6936: seleno-amino acid biosynthesis | 222.91 | 27.7416 |
| PWY-6953: dTDP-3-acetamido-3,6-dideoxy-&alpha;-D-galactose biosynthesis | 0 | 0.20172 |
| PWY-6969: TCA cycle V (2-oxoglutarate:ferredoxin oxidoreductase) | 37.9635 | 26.2213 |
| PWY-7007: methyl ketone biosynthesis | 0 | 0.276332 |
| PWY-7090: UDP-2,3-diacetamido-2,3-dideoxy-&alpha;-D-mannuronate biosynthesis | 0 | 0.171173 |
| PWY-7111: pyruvate fermentation to isobutanol (engineered) | 327.868 | 70.5755 |
| PWY-7115: C4 photosynthetic carbon assimilation cycle, NAD-ME type | 7.94206 | 8.35674 |
| PWY-7117: C4 photosynthetic carbon assimilation cycle, PEPCK type | 21.3302 | 15.7783 |
| PWY-7159: chlorophyllide a biosynthesis III (aerobic, light independent) | 4.96783 | 3.29393 |
| PWY-7184: pyrimidine deoxyribonucleotides de novo biosynthesis I | 116.744 | 56.1879 |
| PWY-7187: pyrimidine deoxyribonucleotides de novo biosynthesis II | 11.9375 | 39.1109 |
| PWY-7197: pyrimidine deoxyribonucleotide phosphorylation | 216.543 | 40.4235 |
| PWY-7198: pyrimidine deoxyribonucleotides de novo biosynthesis IV | 2.8911 | 15.0231 |
| PWY-7199: pyrimidine deoxyribonucleosides salvage | 0.852075 | 3.36211 |
| PWY-7200: superpathway of pyrimidine deoxyribonucleoside salvage | 0 | 1.77803 |
| PWY-7204: pyridoxal 5'-phosphate salvage II (plants) | 0.590365 | 1.50533 |
| PWY-7208: superpathway of pyrimidine nucleobases salvage | 345.491 | 79.9811 |
| PWY-7209: superpathway of pyrimidine ribonucleosides degradation | 0 | 0.133138 |
| PWY-7218: photosynthetic 3-hydroxybutanoate biosynthesis (engineered) | 65.1354 | 23.0255 |
| PWY-7219: adenosine ribonucleotides de novo biosynthesis | 236.501 | 63.7394 |
| PWY-7220: adenosine deoxyribonucleotides de novo biosynthesis II | 214.597 | 94.3318 |
| PWY-7221: guanosine ribonucleotides de novo biosynthesis | 252.359 | 89.6663 |
| PWY-7222: guanosine deoxyribonucleotides de novo biosynthesis II | 214.597 | 94.3318 |
| PWY-7228: superpathway of guanosine nucleotides de novo biosynthesis I | 239.184 | 93.6972 |
| PWY-7229: superpathway of adenosine nucleotides de novo biosynthesis I | 98.2908 | 66.6576 |
| PWY-7234: inosine-5'-phosphate biosynthesis III | 197.177 | 30.5419 |
| PWY-7242: D-fructuronate degradation | 0.517369 | 0.377065 |
| PWY-724: superpathway of L-lysine, L-threonine and L-methionine biosynthesis II | 258.701 | 26.8963 |
| PWY-7254: TCA cycle VII (acetate-producers) | 14.4794 | 12.4226 |
| PWY-7279: aerobic respiration II (cytochrome c) (yeast) | 50.3427 | 47.2161 |
| PWY-7288: fatty acid &beta;-oxidation (peroxisome, yeast) | 1.05467 | 1.76209 |
| PWY-7316: dTDP-N-acetylviosamine biosynthesis | 0 | 1.34152 |
| PWY-7323: superpathway of GDP-mannose-derived O-antigen building blocks biosynthesis | 2.34234 | 3.84566 |
| PWY-7345: superpathway of anaerobic sucrose degradation | 4.30582 | 0 |
| PWY-7371: 1,4-dihydroxy-6-naphthoate biosynthesis II | 0 | 1.00261 |
| PWY-7383: anaerobic energy metabolism (invertebrates, cytosol) | 16.6487 | 12.2278 |
| PWY-7388: octanoyl-[acyl-carrier protein] biosynthesis (mitochondria, yeast) | 3.64079 | 0.803628 |
| PWY-7400: L-arginine biosynthesis IV (archaebacteria) | 203.798 | 23.414 |
| PWY-7539: 6-hydroxymethyl-dihydropterin diphosphate biosynthesis III (Chlamydia) | 8.19966 | 3.34806 |
| PWY-7663: gondoate biosynthesis (anaerobic) | 24.4576 | 29.7661 |
| PWY-7664: oleate biosynthesis IV (anaerobic) | 6.62179 | 1.59199 |
| PWY-821: superpathway of sulfur amino acid biosynthesis (Saccharomyces cerevisiae) | 41.2739 | 39.2905 |
| PWY-841: superpathway of purine nucleotides de novo biosynthesis I | 161.237 | 40.0232 |
| PWY0-1061: superpathway of L-alanine biosynthesis | 54.3787 | 11.5678 |
| PWY0-1241: ADP-L-glycero-&beta;-D-manno-heptose biosynthesis | 6.33549 | 0.986099 |
| PWY0-1261: anhydromuropeptides recycling | 9.97761 | 3.62177 |
| PWY0-1296: purine ribonucleosides degradation | 44.1195 | 14.0044 |
| PWY0-1297: superpathway of purine deoxyribonucleosides degradation | 40.7517 | 7.72353 |
| PWY0-1298: superpathway of pyrimidine deoxyribonucleosides degradation | 0 | 1.37769 |
| PWY0-1319: CDP-diacylglycerol biosynthesis II | 198.046 | 18.2359 |
| PWY0-1479: tRNA processing | 23.0864 | 21.7428 |
| PWY0-1586: peptidoglycan maturation (meso-diaminopimelate containing) | 95.464 | 49.8653 |
| PWY0-162: superpathway of pyrimidine ribonucleotides de novo biosynthesis | 206.378 | 37.0861 |
| PWY0-166: superpathway of pyrimidine deoxyribonucleotides de novo biosynthesis (E. coli) | 114.244 | 53.9009 |
| PWY0-42: 2-methylcitrate cycle I | 5.79835 | 7.70586 |
| PWY0-781: aspartate superpathway | 26.9831 | 11.315 |
| PWY0-845: superpathway of pyridoxal 5'-phosphate biosynthesis and salvage | 0.525284 | 3.17767 |
| PWY0-862: (5Z)-dodec-5-enoate biosynthesis | 31.2615 | 34.3379 |
| PWY490-3: nitrate reduction VI (assimilatory) | 54.6755 | 4.82673 |
| PWY4FS-7: phosphatidylglycerol biosynthesis I (plastidic) | 10.1871 | 10.6645 |
| PWY4FS-8: phosphatidylglycerol biosynthesis II (non-plastidic) | 10.1871 | 10.6645 |
| PWY66-367: ketogenesis | 5.63123 | 7.76279 |
| PWY66-389: phytol degradation | 0 | 11.4664 |
| PWY66-391: fatty acid &beta;-oxidation VI (peroxisome) | 0 | 2.48135 |
| PWY66-398: TCA cycle III (animals) | 34.0519 | 27.8367 |
| PWY66-399: gluconeogenesis III | 13.8841 | 11.5866 |
| PWY66-400: glycolysis VI (metazoan) | 11.5384 | 9.6519 |
| PWY66-409: superpathway of purine nucleotide salvage | 98.963 | 40.0264 |
| PWY66-422: D-galactose degradation V (Leloir pathway) | 0.601707 | 0.528205 |
| PWYG-321: mycolate biosynthesis | 5.18988 | 1.24736 |
| PYRIDNUCSYN-PWY: NAD biosynthesis I (from aspartate) | 48.4923 | 15.5871 |
| PYRIDOXSYN-PWY: pyridoxal 5'-phosphate biosynthesis I | 10.7863 | 19.8298 |
| REDCITCYC: TCA cycle VIII (helicobacter) | 0.553333 | 2.23286 |
| RIBOSYN2-PWY: flavin biosynthesis I (bacteria and plants) | 224.575 | 34.4908 |
| SALVADEHYPOX-PWY: adenosine nucleotides degradation II | 0.498644 | 1.59162 |
| SER-GLYSYN-PWY: superpathway of L-serine and glycine biosynthesis I | 84.1981 | 24.6577 |
| SO4ASSIM-PWY: sulfate reduction I (assimilatory) | 374.692 | 77.2396 |
| SULFATE-CYS-PWY: superpathway of sulfate assimilation and cysteine biosynthesis | 55.0471 | 30.181 |
| TCA-GLYOX-BYPASS: superpathway of glyoxylate bypass and TCA | 19.0181 | 15.1505 |
| TCA: TCA cycle I (prokaryotic) | 57.3643 | 32.8943 |
| TEICHOICACID-PWY: teichoic acid (poly-glycerol) biosynthesis | 0.136099 | 0 |
| THISYN-PWY: superpathway of thiamin diphosphate biosynthesis I | 2.07475 | 4.9356 |
| THRESYN-PWY: superpathway of L-threonine biosynthesis | 123.286 | 30.6368 |
| TRNA-CHARGING-PWY: tRNA charging | 253.679 | 69.6064 |
| TRPSYN-PWY: L-tryptophan biosynthesis | 220.747 | 33.0849 |
| TYRFUMCAT-PWY: L-tyrosine degradation I | 0 | 0.441755 |
| UBISYN-PWY: superpathway of ubiquinol-8 biosynthesis (prokaryotic) | 12.202 | 4.57265 |
| UDPNAGSYN-PWY: UDP-N-acetyl-D-glucosamine biosynthesis I | 246.548 | 18.567 |
| URSIN-PWY: ureide biosynthesis | 0.493903 | 1.25985 |
| VALSYN-PWY: L-valine biosynthesis | 327.868 | 70.5755 |

**Table S2.** List of hydrocarbons (*n*-alkanes, alkenes and isoprenoids, ug·g^-1^ dw) and their stable-carbon isotopic composition (‰) detected in the green and orange endoevaporitic layer samples from Laguna Verde, Argentina.

| Compounds^a^ | **Green** | **Orange** | **Green** | **Orange** |
| --- | --- | --- | --- | --- |
| 14 | 0.01 | 0.01 |  |  |
| 15 | 0.08 | 0.02 |  |  |
| 16 | 0.05 | 0.01 |  |  |
| 17:1 | 0.44 | 0.34 | -31.6 | -32.2 |
| 17 | 0.17 | 0.09 | -29.3 | -29.3 |
| 2,5-di-*n*-alkyltiophenes | 1.27 | 0.42 | -28.6 | -28.9 |
| 18:1 | 2.11 | 0.38 | -28.8 | -27.1 |
| 18 | 0.10 | 0.05 | -28.8 | -29.8 |
| 19:1 | 2.31 | 0.40 |  |  |
| 19 | 0.08 | 0.03 |  |  |
| 20:1 | 0.12 | 0.06 | -26.5 |  |
| 20 | 0.04 | 0.02 | -29.5 |  |
| 21 | 0.10 | 0.05 | -28.6 |  |
| 22:1 | 0.40 | 0.19 | -28.0 | -28.3 |
| 22 | 0.06 | 0.02 | -32.0 | -31.1 |
| 23 | 0.02 | 0.01 |  |  |
| 24 | 0.01 | 0.01 |  |  |
| 25 | 0.03 | 0.01 |  |  |
| 26 | n.d. | n.d. |  |  |
| 27 | 0.13 | 0.02 | -12.8 |  |
| Squalene | 0.20 | 0.16 | -19.7 | -19.9 |
| 28 | n.d. | n.d. |  |  |
| 29 | 0.08 | 0.05 | -20.1 | -21.4 |
| 30 | n.d. | n.d. |  |  |
| 31 | 0.10 | 0.04 | -26.3 | -21.9 |
| Diploptene | 0.19 | 0.01 | -14.7 |  |

n.d. means not detected.

^a^ Linear and saturated (*normal*) alkanes (*n*-alkanes) are named by simple numbers (N) that indicate the number of carbons in the chain; alkanes with a double bond (i.e. alkenes) are named as N:1.

**Table S3.** List of alkanoic acids (ug·g^-1^ dw) detected and their stable-carbon isotopic composition (‰) detected in the green and orange endoevaporitic layer samples from Laguna Verde, Argentina.

| Compounds^a^ | **Green** | **Orange** | **Green** | **Orange** |
| --- | --- | --- | --- | --- |
| 12:0 | 0.06 | 0.06 | -27.4 | -26.7 |
| 13:0 | n.d. | n.d. |  |  |
| *i*14:0 | 0.07 | 0.03 | -19.8 | -21.2 |
| 14:1ω5 | 0.04 | 0.03 | -25.9 | -28.0 |
| 14:0 | 1.60 | 1.46 | -20.5 | -24.7 |
| *i*15:0 | 0.59 | 0.19 | -18.0 | -18.3 |
| a15:0 | 0.60 | 0.23 | -17.6 | -18.5 |
| 15:0 | 0.35 | 0.08 | -18.6 | -20.3 |
| 16:2ω6,9 | 2.27 | 0.97 | -22.8 | -28.7 |
| 16:1ω7 | 37.30 | 6.94 | -19.8 | -22.1 |
| 16:0 | 22.24 | 6.21 | -23.6 | -23.8 |
| MMe-16:0 | 1.68 | 0.26 | -17.5 | -19.9 |
| *i*17:0 | 0.20 | 0.04 | -15.5 | -18.5 |
| a17:0 | 0.29 | 0.10 | -17.7 | -18.7 |
| 17:1ω7 | 0.59 | 0.08 | -23.0 | -24.4 |
| cy17:0 | 0.73 | 0.19 | -17.6 | -20.1 |
| 17:0 | 0.35 | 0.08 | -19.9 | -20.8 |
| 18:5 | 0.32 | 0.05 | -20.4 | -23.5 |
| 18:2ω6,9 | 2.77 | 1.62 | -20.8 | -29.6 |
| 18:1ω9 | 33.34 | 2.16 | -26.5 | -26.0 |
| 18:1ω7 | 6.20 | 1.61 | -27.2 | -23.0 |
| 18:0 | 3.41 | 0.85 | -21.5 | -24.0 |
| MMe-18:0 | 0.24 | 0.07 |  |  |
| 19:1ω9 | 1.30 | 0.22 |  |  |
| cy19:0 | 4.33 | 1.30 | -23.1 | -22.7 |
| 19:0 | 0.08 | 0.01 |  |  |
| 20:4ω3,6,9,12 | 2.90 | 0.24 | -20.0 | -22.5 |
| 20:5ω3,6,9,12,15 | 6.09 | 0.80 | -18.6 | -21.4 |
| 20:1ω9 | 0.55 | n.d. | -23.3 |  |
| 20:0 | 0.57 | 0.09 | -18.7 | -23.6 |
| 21:0 | 0.08 | 0.02 |  |  |
| 22:5ω3,6,9,12,15 | 0.09 | n.d. |  |  |
| 22:6ω3,6,9,12,15,18 | 0.27 | 0.13 | -16.5 | -17.8 |
| 22:0 | 1.11 | 0.29 | -22.9 | -23.8 |
| 23:0 | 0.11 | 0.02 |  |  |
| 24:1ω9 | 1.17 | 0.37 | -19.3 | -19.7 |
| 24:0 | 2.95 | 1.06 | -20.0 | -19.6 |
| 25:0 | 0.11 | 0.03 |  |  |
| 26:1ω9 | 0.26 | 0.05 |  |  |
| 26:0 | 0.35 | 0.08 | -24.9 | -21.4 |

n.d. means not detected.

^a^ The alkanoic acids are named as N:n, where N indicates the total number of carbon in the chain and n the number of double bonds. In polyunsaturated acids, the positions of the double bonds are indicated in the omega notation (i.e., respect to the end carbon). *Iso* (*i*N:0) and *anteiso* (*a*N:0) alkanoic acids are alkanoic acids with a methyl group in position N-1 or N-2, respectively. Other branched alkanoic acids include mono- (MMe), methylated chains.

**Table S4.** List of alcohols and sterols detected acids (ug·g^-1^ dw) detected and their stable-carbon isotopic composition (‰) detected in the green and orange endoevaporitic layer samples from Laguna Verde, Argentina.

| Compounds^a^ | **Green** | **Orange** | **Green** | **Orange** |
| --- | --- | --- | --- | --- |
| 14 | 0.003 | 0.001 |  |  |
| 15 | n.d. | 0.003 |  |  |
| 16:1 | 0.079 | 0.015 | -24.8 |  |
| 16 | 0.054 | 0.032 | -18.7 | -19.9 |
| 17 | 0.006 | n.d. | -22.3 |  |
| 18:1 | 0.044 | 0.007 | -19.3 |  |
| 18 | 0.036 | 0.022 | -28.0 | -30.1 |
| 19:1 | 0.006 | n.d. | -27.1 |  |
| 19 | n.d. | n.d. |  |  |
| 20 | 0.016 | 0.008 | -25.4 | -27.6 |
| 22 | 0.007 | n.d. | -29.0 |  |
| Isophytol | 0.521 | n.d. | -24.4 |  |
| Neophytadiene | 0.326 | 0.125 | -25.2 | -23.2 |
| Phytol | 0.822 | n.d. | -24.6 |  |
| Dihydrophytol | 0.014 | 0.008 | -21.0 | -16.9 |
| Cholesterol | 0.114 | n.d. | -16.1 |  |
| Campesterol | 0.029 | n.d. | -18.3 |  |
| β-Sitosterol | 0.013 | n.d. | -18.4 |  |
| Stigmastanol | 0.014 | n.d. | -16.2 |  |

n.d. means not detected.

^a^ Linear and saturated (*normal*) alkanols (*n*-alkanols) are named by simple numbers (N) that indicate the number of carbons in the chain; alkanols with a double bond are named as N:1.
